# Microstructural integrity of the locus coeruleus and its tracts reflect noradrenergic degeneration in Alzheimer’s disease and Parkinson’s disease

**DOI:** 10.1186/s40035-024-00400-5

**Published:** 2024-02-09

**Authors:** Chen-Pei Lin, Irene Frigerio, John G. J. M. Bol, Maud M. A. Bouwman, Alex J. Wesseling, Martin J. Dahl, Annemieke J. M. Rozemuller, Ysbrand D. van der Werf, Petra J. W. Pouwels, Wilma D. J. van de Berg, Laura E. Jonkman

**Affiliations:** 1 Amsterdam UMC, Department of Anatomy and Neurosciences, Location Vrije Universiteit Amsterdam, De Boelelaan 1117, 1081 HV Amsterdam, The Netherlands; 2 Amsterdam Neuroscience, Brain imaging, Amsterdam, The Netherlands; 3 Center for Lifespan Psychology, Max Planck Institute for Human Development, 14195 Berlin, Germany; 4 Leonard Davis School of Gerontology, University of Southern California, Los Angeles, CA 90089 USA; 5 Amsterdam UMC, Department of Pathology, Location Vrije Universiteit Amsterdam, De Boelelaan 1117, Amsterdam, The Netherlands; 6 Amsterdam Neuroscience, Neurodegeneration, Amsterdam, The Netherlands; 7 Amsterdam Neuroscience, Compulsivity, Impulsivity and Attention Program, Amsterdam, The Netherlands; 8 Amsterdam UMC, Department of Radiology and Nuclear Medicine, Location Vrije Universiteit Amsterdam, De Boelelaan 1117, Amsterdam, The Netherlands

**Keywords:** Locus coeruleus, Noradrenergic degeneration, Alzheimer’s disease, Parkinson’s disease, Diffusion MRI, Post-mortem, Histopathology

## Abstract

**Background:**

Degeneration of the locus coeruleus (LC) noradrenergic system contributes to clinical symptoms in Alzheimer’s disease (AD) and Parkinson’s disease (PD). Diffusion magnetic resonance imaging (MRI) has the potential to evaluate the integrity of the LC noradrenergic system. The aim of the current study was to determine whether the diffusion MRI-measured integrity of the LC and its tracts are sensitive to noradrenergic degeneration in AD and PD.

**Methods:**

Post-mortem in situ T1-weighted and multi-shell diffusion MRI was performed for 9 AD, 14 PD, and 8 control brain donors. Fractional anisotropy (FA) and mean diffusivity were derived from the LC, and from tracts between the LC and the anterior cingulate cortex, the dorsolateral prefrontal cortex (DLPFC), the primary motor cortex (M1) or the hippocampus. Brain tissue sections of the LC and cortical regions were obtained and immunostained for dopamine-beta hydroxylase (DBH) to quantify noradrenergic cell density and fiber load. Group comparisons and correlations between outcome measures were performed using linear regression and partial correlations.

**Results:**

The AD and PD cases showed loss of LC noradrenergic cells and fibers. In the cortex, the AD cases showed increased DBH + immunoreactivity in the DLPFC compared to PD cases and controls, while PD cases showed reduced DBH + immunoreactivity in the M1 compared to controls. Higher FA within the LC was found for AD, which was correlated with loss of noradrenergic cells and fibers in the LC. Increased FA of the LC-DLPFC tract was correlated with LC noradrenergic fiber loss in the combined AD and control group, whereas the increased FA of the LC-M1 tract was correlated with LC noradrenergic neuronal loss in the combined PD and control group. The tract alterations were not correlated with cortical DBH + immunoreactivity.

**Conclusions:**

In AD and PD, the diffusion MRI-detected alterations within the LC and its tracts to the DLPFC and the M1 were associated with local noradrenergic neuronal loss within the LC, rather than noradrenergic changes in the cortex.

**Supplementary Information:**

The online version contains supplementary material available at 10.1186/s40035-024-00400-5.

## Background

The noradrenergic system, ascending from the locus coeruleus (LC) and projecting to limbic and cortical brain regions [1], supports cognitive processes including attention, memory and executive functions via the release of noradrenaline [2–4]. Dysregulation of the LC -noradrenergic system leads to deterioration of the aforementioned cognitive functions [5] and manifests in the pathophysiology of both Alzheimer’s disease (AD) and Parkinson’s disease (PD) [2, 4]. Restoring the LC-noradrenergic system has thus become an important target in both diseases [6–8], and neuroimaging biomarkers assaying the LC-noradrenergic system for monitoring disease progression and potential treatment effects are needed [9, 10].

Magnetic resonance imaging (MRI) tools assessing the integrity of the LC are continuously in development [10, 11]. For instance, LC-sensitive imaging showed reduced LC signal intensity in both AD and PD patients compared to controls [12–16]. Although the biophysiological interpretation of the LC signals requires further investigation [17, 18], assessments of LC integrity have shown potentials for patient stratification and predicting a noradrenergic treatment response in relation to behavioral improvements [19, 20]. Another MRI technique, diffusion MRI, is able to assess the microstructural integrity of the white and grey matter with its derivatives, fractional anisotropy (FA) and mean diffusivity (MD) [21–26]. Increased LC FA and decreased MD in the aging population compared to young controls, as well as their association with a decline in memory performance, highlight the capability of diffusion MRI to capture LC microstructural integrity [26]. Furthermore, a recent double-blind randomized three-way crossover study showed great potential of diffusion MRI markers for patient stratification: they identified likely responders from non-responders to noradrenergic treatments in PD with an accuracy of 77%–79% [27]. In a randomized, double-blind placebo-controlled crossover study, PD patients administered with atomoxetine showed increased FA in white matter tracts, which was associated with improved response inhibition [28], suggesting that diffusion measures assessing the integrity of white matter tracts may be a potential predictor of behavioral recovery. Altogether, these findings suggest that developing and validating diffusion MRI markers targeting the LC-noradrenergic system are beneficial for assessing noradrenergic treatment effects in AD and PD.

Profound LC-noradrenergic neuronal and fiber loss, as well as neuropathological burdens of hyperphosphorylated-tau (p-tau), amyloid-beta (Aβ) and alpha-synuclein (α-syn), have been described in AD and PD, accompanied by reduced cortical noradrenergic innervation and in turn reduced cortical noradrenaline levels [29, 30]. The vulnerability of noradrenergic axons stems from their nature—they are long and highly branched with less myelination that allows noradrenergic innervation and efficient neurotransmission in the neocortex. In AD, this diminished cortical noradrenaline, especially in the hippocampus, has been associated with memory deficits [31]. In PD and PD dementia (PDD), a reduction in noradrenergic fibers has been shown in the primary motor cortex (M1) compared to controls [32], suggesting an involvement of noradrenergic denervation in motor deficits [33]. In addition, cognitive impairment has been linked to reduced noradrenaline levels in the prefrontal cortex (PFC) and anterior cingulate cortex (ACC) in both PD and PDD [7, 34]. These postmortem studies suggest that degeneration of tracts from the noradrenergic LC to the hippocampus, PFC, ACC and M1 may contribute to cognitive and/or motor symptoms in AD and PD(D). Despite the noradrenergic system being implicated in AD and PD, currently, only a limited number of studies have investigated the in vivo integrity of the LC-to-cortex tracts in AD and controls [25, 35, 36], and no research has been done in PD. Moreover, no studies have validated their results with the ground truth of human LC-noradrenergic neuronal and fiber loss.

We previously developed a pipeline for post-mortem in situ MRI in combination with immunohistochemistry to study the correlation between MRI measures and neuronal markers in the same brain donor(s) [37]. Here, we used this pipeline to study whether diffusion MRI measures of microstructural alterations within the LC and the cortical projections from LC reflect the severity of LC-noradrenergic degeneration in AD and PD. We hypothesized that: (i) AD and PD cases show altered diffusion MRI measures in the LC and its projections to the PFC, ACC, M1 and hippocampus, (ii) these MRI-measured alteration(s) correlate with LC-noradrenergic neuronal loss and/or reduced noradrenergic innervation in the cortex. This study may aid the development of noradrenergic-sensitive in vivo MRI markers for assessing the degeneration of the LC-noradrenergic system.

## Methods

### Donor inclusion

**Fig. 1:**
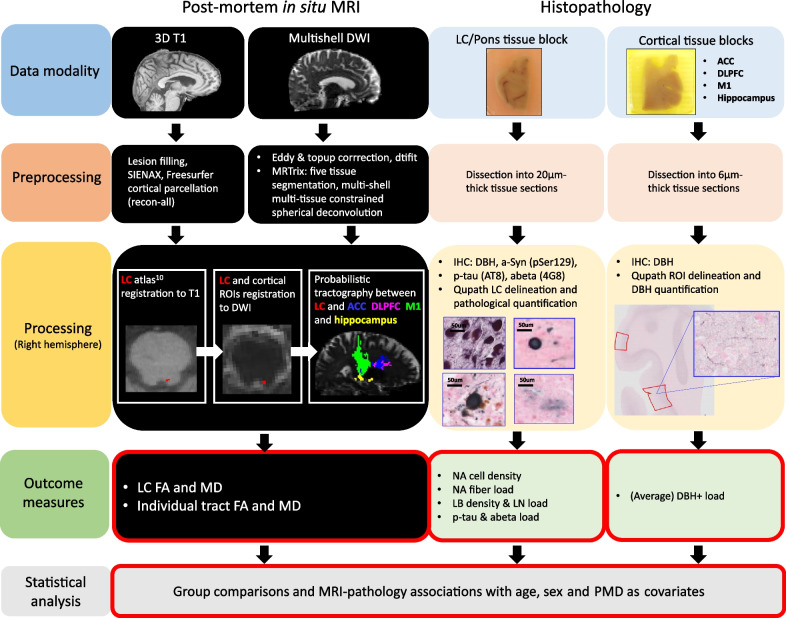
Flow chart of the study. After donor inclusion, post-mortem in situ 3D T1 and multi-shell diffusion MRI images were collected and (pre)processed: the LC was segmented [44] and the LC tracts to the anterior cingulate cortex (ACC, blue tract), the dorsolateral prefrontal cortex (DLPFC, purple tract), the primary motor cortex (M1, green tract) and the hippocampus (yellow tract) were reconstructed, deriving the FA and the MD of the LC and its tracts. Autopsy and brain dissection were performed after the in situ MRI scans. Brain tissue blocks were formalin-fixated for 4 weeks, then dissected and paraffin-embedded. Next, 20-µm-thick sections were processed for immunohistochemistry for dopamine-beta hydroxylase (DBH), phosphorylated Ser129 α-synuclein (pSer129-αsyn), phosphorylated-tau (p-tau) and amyloid-β (Aβ), and imaged using a whole-slide scanner. Immunoreactivity in regions of interest were analyzed using Qupath, deriving the LC-noradrenergic cell density and fiber load, Lewy body (LB) density and Lewy neurite (LN) load. The group comparisons between AD, PD and controls were investigated with general linear models and the association between MRI and pathology markers was investigated with linear regression models, including age, sex and post-mortem delay as covariates. Abbreviations: DWI, diffusion-weighted tensor imaging; LC, locus coeruleus; NA, noradrenergic; IHC, immunohistochemistry; FA, fractional anisotropy; MD, mean diffusivity; LB, Lewy body; LN, Lewy neurite; PMD, post-mortem delay

A total of 31 clinically defined and pathologically confirmed brain donors were included in the current post-mortem MRI and pathology study: 9 amnestic AD, 14 PD, as well as 8 age- and sex-matched control donors. During life, all donors provided written informed consent for the use of their brain tissue and medical records for research purposes. AD and PD donors were included in collaboration with the Netherlands Brain Bank (NBB; http://brainbank.nl). PD and PDD were diagnosed based on clinical presentations according to the Movement Disorder Society clinical diagnostic criteria [38–40]. Disease durations were extracted from the clinical files of the donors. The control donors were included at the Department of Anatomy and Neurosciences, Amsterdam UMC, following the Normal Aging Brain Collection Amsterdam (NABCA; http://nabca.eu) pipeline [37]. All donors underwent post-mortem in situ MRI with subsequent brain autopsy and dissection of brainstem, limbic and cortical regions based on the Brain Net Europe (BNE) and NABCA protocol [37, 41]. Neuropathological diagnosis was performed by an expert neuropathologist (AJMR) according to the international guidelines of BNE. The study design is summarized in Fig. 1.

### MRI acquisition

Post-mortem MRI data were acquired through in situ (brain still in cranium) scanning on a whole-body 3T MR scanner (Signa-MR750, General Electric Medical Systems, Chicago, IL) with an eight-channel phased-array head-coil [37]. T1-weighted images (T1w) were acquired using a sagittal 3D T1-weighted fast spoiled gradient echo sequence (FSPGR) with the following parameters: repetition time (TR)/echo time (TE)/inversion time (TI) = 7/3/450 ms, flip angle = 15°, slice thickness = 1 mm, in-plane resolution = 1.0 × 1.0 mm^2^. A sagittal 3D fluid attenuation inversion recovery (FLAIR) sequence was acquired with TR/TE/TI = 8000/130/2000–2250 ms, slice thickness 1.2 mm, and in-plane resolution = 1.11 × 1.11 mm^2^. In addition, the inversion time of the FLAIR sequence was optimized per case to account for variable CSF suppression due to post-mortem delay (PMD; time between death and MRI scanning). Diffusion-weighted images (DWI) were acquired with a multi-shell single-spin echo-planar imaging sequence (TR = 7651 ms, TE = 104 ms, 1.75 × 1.75 mm^2^ in-plane resolution, slice thickness 2 mm) with 89 interleaved directions (30 *b* = 1000 s/mm^2^ and 59 *b* = 2000s/mm^2^) and 6 non-diffusion-weighted volumes (*b* = 0 s/mm^2^). To allow for geometric distortion correction, b0 volumes with reversed phase-encode direction were obtained.

### MRI analysis

#### Structural image processing and LC atlas registration

To minimize the impact of age-related white matter abnormalities (e.g., vascular change) on automated segmentations, the 3D T1 images were lesion-filled [42], as previously described. Subsequently, normalized brain volumes of the whole brain, white matter, and gray matter were estimated from 3D T1 images using SIENAX [43], FMRIB Software Library (FSL) tools version 6.0.4 (https://fsl.fmrib.ox.ac.uk/fsl/). To delineate the LC of each case, an existing binary LC atlas of the entire LC from Dahl et al*.* [11, 44] was used. First, for each case, the whole brain was skull-stripped and co-registered to standard space (MNI-ICBM 152 linear, 0.5 mm) using a template-based procedure implemented in Advanced Normalization Tools (ANTs, version 2.1) [45, 46]. The inverse of the transformation matrices was then applied to the LC meta mask to warp it to native space with nearest neighbor interpolation, deriving the entire LC in subject T1 space. The complete script can be found in the Additional file 1. In addition, to derive the cortical regions of interest (ROIs), namely the ACC, dorsolateral prefrontal cortex (DLPFC), M1 and hippocampus, each hemisphere was parcellated into 34 anatomical cortical regions using the Desikan–Killany atlas with Freesurfer, version 7.0 (http://surfer.nmr.mgh.harvard.edu) [47]. The reconstructed datasets were visually inspected, and segmentation errors were corrected. The LC and cortical ROIs of individual brain scans were transformed from 3D T1 to diffusion space: the DWI images were first co-registered to 3D T1 to generate the transformation matrices, which were inverted and used for registering the ROIs to diffusion space for further probabilistic tractography [48]. LC registration from the atlas to diffusion space is illustrated in Additional file 1: Fig. S1. All cases were visually inspected for the location of the LC in both 3D T1 and DWI, from the dorsal end of the inferior colliculus to the floor of fourth ventricle, to ensure its correct anatomical location.

#### DWI pre-processing, microstructural assessment and probabilistic tractography

DWI was first denoised using the dwidenoise tool in MRtrix3, to improve the generally low signal-to-noise ratio in DWI [49]. Subsequently, the susceptibility-induced off-resonance field was estimated and corrected from pairs of images with opposite phase-encoding directions using topup, and eddy current-induced distortion correction was applied with the FSL software suite [50]. To assess the microstructural integrity of the LC and its tracts, we fitted the tensor to *b* = 2000 s/mm^2^ data to determine FA and MD using FSL DTIFIT [51]. FA and MD of *b* = 1000 s/mm^2^ data were also fitted for validation. The FA and MD maps of both shells were registered to 3D T1 with trilinear interpolation, and average values of the LC were extracted in subject T1 space.

To assess the LC tract microstructural integrity, we reconstructed the LC tracts in the right hemisphere, as the tissue blocks and subsequent pathological outcome measures were only available from this hemisphere. Tractography was performed with MRtrix3, using anatomically-constrained probabilistic tractography (ACT). To achieve this, 3D T1 images were segmented based on five-tissue response functions, and transformed to DWI space, providing information on streamline propagation and termination during tractography. In addition, the fiber orientation in each voxel was estimated with Multi-Shell Multi-Tissue Constrained Spherical Deconvolution (MSMT-CSD) [52, 53]. Embedded within the tractography function (tckgen), ACT was performed with the LC as the seed ROI and the cortical ROIs (ACC, DLPFC, M1, and hippocampus) as separate target ROIs, resulting in four tracts: LC-ACC, LC-DLPFC, LC-M1 and LC-hippocampus, with 5000 streamlines per tract with a maximum length of 250 mm. In addition, to ensure that streamlines are within the right hemisphere, a left hemisphere mask was generated, and used as an exclusion mask during ACT. Thereafter, the FA and MD of individual tracts were determined based on the probability-weighted mean along the tract.

### Histology and immunohistochemistry processing

#### Tissue sampling

After post-mortem in situ MRI, donors were transported to the mortuary for craniotomy. The left hemisphere was instantly dissected and snap-frozen in liquid nitrogen, and stored for molecular and biochemical analysis. The right hemisphere was fixed in 4% formalin for four weeks. Subsequently, the right hemisphere was dissected based on the BNE sampling protocol to extract the LC, ACC, DLPFC, M1 and hippocampus (including the entorhinal cortex, parahippocampal and fusiform gyrus, as described previously [54, 55]). The rostral LC neurons project to the forebrain regions, hippocampus and septum; whereas the middle and caudal portions of the LC project to the cerebellum, basal ganglia and spinal cord [56, 57]. Therefore, the rostral LC was included in the current study in order to examine the noradrenergic projections to the ACC, DLPFC, M1 and hippocampus. All tissue blocks were paraffin-embedded and stained with (immuno)histochemistry [37, 41].

#### Immunohistochemistry

For detailed methods, see Additional file 1. In brief, paraffin-embedded tissue blocks of the LC were cut into 4 × 20 µm-thick consecutive sections, and stained with monoclonal rabbit anti-dopamine-beta hydroxylase (DBH, dilution 1:400, Abcam, Cambridge, UK), rabbit anti-phosphorylated-Ser129 α-syn (clone EP1536Y, dilution 1:4000, Abcam), mouse anti-p-tau (clone AT8, dilution 1:800, ThermoFisher, Pittsburgh, PA) or mouse anti-Aβ (clone 4G8, dilution 1:5000, BioLegend, San Diego, CA) antibody. The paraffin-embedded cortical tissue blocks, the ACC, DLPFC, M1 and hippocampus, were cut at 6 µm and processed for immunohistochemistry with rabbit anti-DBH (dilution 1:400, Abcam). The DLPFC sections were stained with an alternative DBH antibody for validation (dilution 1:100, Novus, Cambridge, UK, Additional file 1). The primary antibodies were incubated at 4°C overnight and visualized with Vector SG grey (Vector, Newark, CA), 3,3′-diaminovenzidine (DAB, Sigma-Aldrich, Darmstadt, Germany), or DAB + Nickel, followed by counter-staining with nuclear fast red (Vector). The sections were dehydrated in a series of ethanol, xylene, and then mounted with Entellan.

#### ROI delineation and pathological quantification

Immunostained sections of the LC were digitally scanned with the Vectra Polaris Quantitative Pathology Imaging System (PerkinElmer, Waltham, MA) at 200 × magnification with a 20 × objective. Immunostained sections of the cortical regions were scanned with Olympus VS200 (Evident, Japan) using a focus map grid to capture the DBH-stained fibers. Qupath Software Version 0.2.3 [58] was used to process the scanned images, perform the delineation of the LC and cortical regions, and finally quantify the noradrenergic neurons and fibers, as well as α-syn, p-tau and Aβ pathological load [58]. Based on a previously described delineation method [56, 59, 60], the LC delineation was defined by placing a 2.5 mm^2^ sampling grid at the center of the cluster of neuromelanin-containing cells on DBH-stained sections. With the aid of neighboring anatomical landmarks, namely the fourth ventricle, mesencephalic trigeminal nucleus and its tract, and superior cerebellar peduncle [30], the sampling grid was placed at a similar level of the LC in each case, independent of the neuromelanin-containing cell load (Additional file 1: Fig. S2). The same sampling grid was transferred to consecutive stained sections of α-syn, p-tau and Aβ. Noradrenergic neurons, represented by DBH-positive (DBH^+^) neurons, and immunoreactivity of α-syn, p-tau and Aβ, were quantified using the in-house QuPath scripts. For cortical regions stained with DBH, ROIs containing all cortical layers were delineated in straight areas of the cortex, to avoid over- or under-estimation of immunoreactivity in sulci and gyri, respectively [61, 62]. Hippocampal sections were segmented according to the method described by Adler et al., in which CA1 to CA4 regions were included and combined [55]. In cortical regions, we quantified structures with highly intense DBH^+^ signals that form elongated, as well as dot-like structures, representing the traveling and cross-sectional noradrenergic axons, respectively (Additional file 1: Fig. S3). These intense signals inevitably include few punctate staining of DBH, we thus took the outcome of DBH quantification as DBH^+^ load (%).

Detailed description of the quantification can be found in Additional file 1: Methods and Figs. S2 and S3. To summarize, the immunohistochemistry outcome measures included were DBH-positive (DBH^+^) noradrenergic cell density (count/mm^2^), noradrenergic fiber load (% area), Lewy body (LB) density (count/mm^2^), Lewy neurite (LN) load (% area), p-tau load (% area) and Aβ load (% area) within the LC, as well as the DBH^+^ load (% area) within each cortical area.

### Statistical analysis

Statistical analysis was performed using IBM SPSS 22.0 for Windows (SPSS, Inc., Chicago, IL). All statistical variables were tested for normality. Chi-square tests were used to analyze group difference between AD, PD and control donors for categorical variables. General linear models (GLM) were used for the aforementioned group differences in MRI-derived outcome measures (FA and MD of LC, as well as FA and MD of individual tracts), and histopathological outcome measures. We applied linear regression models to examine the associations between the above-mentioned MRI and histopathology-derived outcome measures. Age, sex and PMD were included as covariates in all analysis. The regression models were performed in two ways: (1) within the whole cohort; (2) only within the control + AD or control + PD groups. Group comparisons and correlation analysis were followed by false discovery rate (FDR) correction for multiple comparisons [63]. Graphical illustrations were made with Biorender (https://biorender.com/), R (https://www.rstudio.com/) and Graphpad (https://www.graphpad.com/). Since our PD group included PD and PDD, for the clarity purpose, we labeled PD in dark blue and PDD in light blue in figures.

## Results

### Clinical, radiological and pathological characteristics

Group demographics are summarized in Table 1. For detailed donor information, see Additional file 1: Table S1. For clinical characteristics, compared to controls, no differences in age, sex or PMD were found for AD and PD cases. Compared to AD, PD cases were older (*P* = 0.028) and had a longer disease duration (*P* = 0.012). For the radiological characteristics of normalized total grey matter volume, the AD group showed a lower volume compared to the PD and control groups (*P* = 0.004 and *P* = 0.013, respectively), while no differences were found between PD and control cases. No difference was found for normalized total brain or white matter volume. For pathological characteristics, the AD cases showed higher Thal and Braak stages of neurofibrillary tangle compared to both PD and control cases (both *P* < 0.001), while no difference was found between PD and control cases. All PD cases had Braak LB stage 6, while both AD and control cases had Braak LB stage 0.

**Table 1:** Demographics, clinical, radiological and pathological characteristics of included cohort

	Control	AD	PD
*Clinical characteristics*			
Number of cases	8	9	14 (8 PD and 6 PDD)
Sex, male/female (%male)	5/3 (63%)	7/2 (78%)	10/4 (71%)
Age at death, years	73 ± 11 (range 57–85)	64 ± 10 (53–84)^**#**^	75 ± 6 (62–90)
Disease duration, years	−	9 ± 6^#^	15 ± 4
Post-mortem delay (h:min)	7:18 ± 2:14	8:12 ± 0:58	7:06 ± 1:52
*Radiological characteristics*			
Normalized brain volume (ml)	1468.7 ± 69.1	1391.8 ± 132.4	1417.9 ± 90.1
Normalized grey matter volume (ml)	732.4 ± 52.9	649.2 ± 64.6*,^**#**^	700.6 ± 47.0
Normalized white matter volume (ml)	736.4 ± 37.8	742.7 ± 105.8	717.4 ± 73.5
*Pathological characteristics*			
Thal phase (*n*)	8	9^**,##^	14
0/1/2/3/4/5	1/6/0/0/1/0	0/0/0/1/4/4	0/7/3/4/0/0
Braak neurofibrillary tangle stage (*n*)	8	9^**,##^	14
0/I/II/III/IV/V/VI	2/2/1/3/0/0/0	0/0/0/0/1/5/3	0/1/10/3/0/0/0
Braak Lewy body stage (*n*)	8	9	14
0/1/2/3/4/5/6	8/0/0/0/0/0/0	9/0/0/0/0/0/0	0/0/0/0/0/0/14

Mean ± SD; h, hour; min, minutes

*P < 0.05 compared to controls

**P < 0.001 compared to controls

^#^*P* < 0.05 compared to PD

^##^*P* < 0.001 compared to PD

### Microstructural integrity of the LC and its tracts in AD and PD

**Fig. 2:**
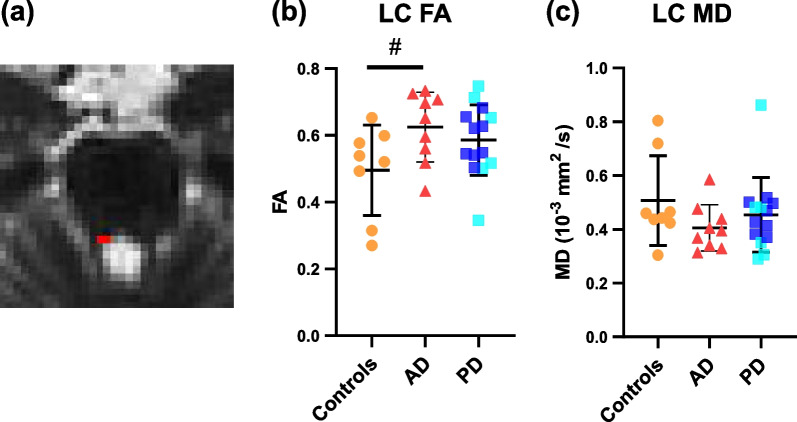
LC microstructural integrity in controls, AD and PD. **a** The LC (red) of the right hemisphere in DWI in a radiological view.** b** Increased LC FA (*P* = 0.040, uncorrected) was found in AD cases compared to controls. **c** No significant difference in LC MD was found between groups. Within the PD group, PD cases are labeled with darker blue, whereas PDD cases are labeled with lighter blue. ^#^*P* < 0.05, uncorrected

The average LC volumes in both T1 and DWI space (before normalization to the intracranial brain volume) in each group can be found in Additional file 1: Table S2. The resulting LC masks are LC-enriched, as the LC volumes were slightly larger than LC volume estimation in stereological studies [56, 64]. On T1 MRI, the LC volume did not differ between groups (AD: 26.78 ± 7.33 mm^3^, PD: 25.12 ± 8.85 mm^3^, controls: 26.88 ± 5.65 mm^3^). On diffusion MRI, the AD group showed higher FA compared to controls (*P* = 0.040, uncorrected, Fig. 2a), though this significance did not survive correction for multiple comparisons. No difference in FA was found between PD and controls or between AD and PD. The MD showed no difference between groups (Fig. 2). FA and MD of the LC extracted from *b* = 1000 shell showed similar results; a higher FA of the LC (trend-level) was found in AD compared to controls, while no differences were found between PD and controls. No group difference was found for MD (Additional file 1: Table S3). As for the tracts between the LC and cortical regions, no significant group differences were found in either FA or MD (Additional file 1: Table S4).

### Severity of LC neuronal loss and pathological burden in AD and PD

**Fig. 3:**
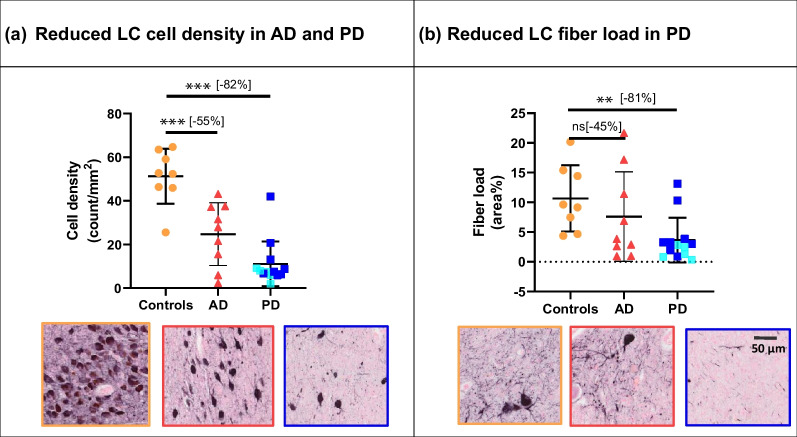
Noradrenergic cell density and fiber load within the LC of controls, AD and PD cases. **a** Noradrenergic cell density was lower in AD and PD compared to control cases. **b** Noradrenergic fiber load was lower in PD compared to control cases. Scale bar, 50 µm for all images. Within the PD group, PD cases are labeled with darker blue, whereas PDD cases are labeled with lighter blue. ns, not significant. **P < 0.01 FDR-corrected, ***P < 0.001 FDR-corrected

Both the AD and the PD cases showed noradrenergic neuronal loss within the LC (Fig. 3). Compared to controls, the AD cases showed a 55% decrease of noradrenergic cell density (*P* = 0.0002, FDR-corrected) and a trend of noradrenergic fiber loss (− 45%, *P* = 0.067, uncorrected). In turn, the PD cases showed a 82% decrease of noradrenergic cell density (*P* < 0.0001, FDR-corrected) and a 81% decrease of noradrenergic fiber load (*P* = 0.003, FDR-corrected), compared to controls. Compared to the AD cases, the PD cases showed a trend of reduced noradrenergic cell density (− 61%, *P* = 0.051, uncorrected), but no significant difference in noradrenergic fiber load (*P* = 0.124) (Fig. 3). Both AD and control cases showed a large within-group variability in LC noradrenergic cell density and fiber loads, whereas PD cases showed less within-group variability (Fig. 3).

With regard to the pathological burden within the LC, both Aβ and p-tau pathology were found in the AD cases, while only some p-tau pathology was found in PD and control cases (Additional file 1: Fig. S4). α-Syn pathology in the forms of LB and LN was found in PD cases and not in controls. Only one AD case showed a LB in the LC (Additional file 1: Fig. S5), but did not meet the criteria of Braak LB stage 1. We found no correlations between noradrenergic loss within the LC and the pathological burden in AD or PD (Additional file 1: Table S5). We also examined the association with disease duration but found no correlations for noradrenergic cell density or fiber load (Additional file 1: Table S6). The AD group consisted of early- and late-onset (EOAD and LOAD) cases. The EOAD cases carrying homozygous *APOE*4 had more severe neuronal loss (Additional file 1: Fig. S6).

### Reduced cortical noradrenergic innervation in PD, but increased innervation in AD

The noradrenergic innervation of the ACC, DLPFC, M1 and hippocampus was compared between groups (Additional file 1: Fig. S7). Noradrenergic fibers were identified based on an intense DBH^+^ staining and varicosities (shape of bouton) along the axons, which were present in both deep and superficial layers of the cortex. Consistent with previous literature [65], we observed relatively weak DBH staining in dendritic, somatic and axonal synapses (punctate staining as shown in Additional file 1: Fig. S3). In the ACC, M1 and hippocampus, more elongated noradrenergic fibers were observed in both superficial and deep cortical layers of controls compared to AD and PD donors. In addition, AD and PD donors showed more fibers with pathological features such as larger varicosities along the axons, and tangled or clustered fiber structures. In the DLPFC, surprisingly, we observed increased noradrenergic fibers and (synaptic) innervation in both deep and superficial cortical layers of the AD donors. The staining patterns resembled sprouting fibers and synaptic staining surrounding the soma of cortical neurons (Additional file 1: Fig. S8). This observation was confirmed with another commercial antibody polyclonal rabbit anti-DBH (dilution 1:100, Novus, Cambridge, UK; Additional file 1: Methods, Fig. S9).

**Fig. 4:**
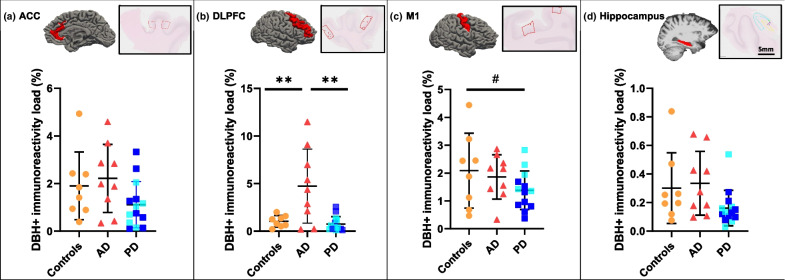
Noradrenergic innervation in the ACC, DLPFC, M1 and hippocampus. **a** PD showed a trend of reduced DBH^+^ load in the ACC compared to AD (*P* = 0.061, uncorrected), while no difference was found between AD and controls. **b** AD showed significantly increased DBH^+^ load in the DLPFC compared to both PD and control donors (respectively, *P* = 0.006 and *P* = 0.007, both FDR-corrected), while no difference was found between PD and controls. **c** PD showed reduced DBH^+^ load in the M1 compared to controls (*P* = 0.03, uncorrected), while no difference was found between AD and PD or between AD and controls. **d** No group differences in the DBH^+^ load in the hippocampus was found. Within the PD group, PD cases are labeled with darker blue, whereas PDD cases are labeled with lighter blue. Scale bar, 5 mm for all images. ^#^*P* < 0.05, uncorrected; **P < 0.01, FDR-corrected

On a statistical level, the PD cases showed a 45% decrease of DBH^+^ load in the ACC compared to AD (*P* = 0.061, uncorrected, Fig. 4a), and a 34% decrease of DBH^+^ load in the M1 compared to controls (*P* = 0.03, uncorrected, Fig. 4c). No group differences were found between AD and controls in these two regions (Fig. 4). For the hippocampus, no group differences were found between any pair of groups (Fig. 4d). In the DLPFC, as expected from the descriptive results of DBH-immunoreactivity, the AD cases showed higher DBH^+^ load compared to controls and PD (increase by 78% and 87%, respectively, *P* = 0.006 and *P* = 0.007, both FDR-corrected), while no difference was found between PD and controls (Fig. 4b). As a previous study indicated an age effect on the degeneration of the noradrenergic cells and fibers in the LC and cortical ROIs [66], we also examined the correlation between age and DBH-immunoreactivity in the LC and all cortical regions, and found no age effect in our cohort (Additional file 1: Table S7).

### Changes in cortical noradrenergic innervation are associated with LC neuronal loss in AD and PD

Since we found that the DLPFC and M1 showed altered noradrenergic innervation in AD and PD, respectively, we hypothesized that these alterations may stem from noradrenergic cell and/or fiber loss within the LC. We found that the increased DBH^+^ load in the DLPFC was correlated with decreased LC noradrenergic cell density in the combined AD and control group (*r* = -0.52, *P* = 0.036, FDR-corrected), but not in the AD group alone. In the M1 region, trend-level correlations were found between reduced DBH^+^ load in the M1 and reduced LC noradrenergic cell density in the combined PD and control group (*r* = 0.47, *P* = 0.063 uncorrected), as well as between reduced DBH^+^ load and increased LC noradrenergic fiber load in the PD group only (*r* = 0.50, *P* = 0.071 uncorrected) (Table 2).

**Table 2:** Correlations of LC noradrenergic cell density and fiber load with DBH^+^ load in the DLPFC and M1

	DLPFC DBH^+^ load (%)	
	Pearson's* r*	*P* value
*AD combined with controls*		
LC NA cell density (counts/mm^2^)	** − 0.515**	0,036*
LC NA fiber load (%)	** − **0,036	0,454
*AD*		
LC NA cell density (counts/mm^2^)	** − **0.406	0.212
LC NA fiber load (%)	0.028	0.479

	M1 DBH^+^ load (%)	
	Pearson's* r*	*P* value
*PD combined with controls*		
LC NA cell density (counts/mm^2^)	**0.468**	**0.063**
LC NA fiber load (%)	0.376	0.114
*PD*		
LC NA cell density (counts/mm^2^)	** − **0.157	0.332
LC NA fiber load (%)	**0.498**	**0.071**

*AD* Alzheimer’s disease, *PD* Parkinson’s disease, *LC* locus coeruleus, *NA* noradrenergic, *DBH* dopamine-beta hydroxylase, *DLPFC* dorsolateral prefrontal cortex, *M1* primary motor cortex

### Diffusion MRI-measured alterations in the LC and its tracts reflect LC neuronal loss

**Fig. 5:**
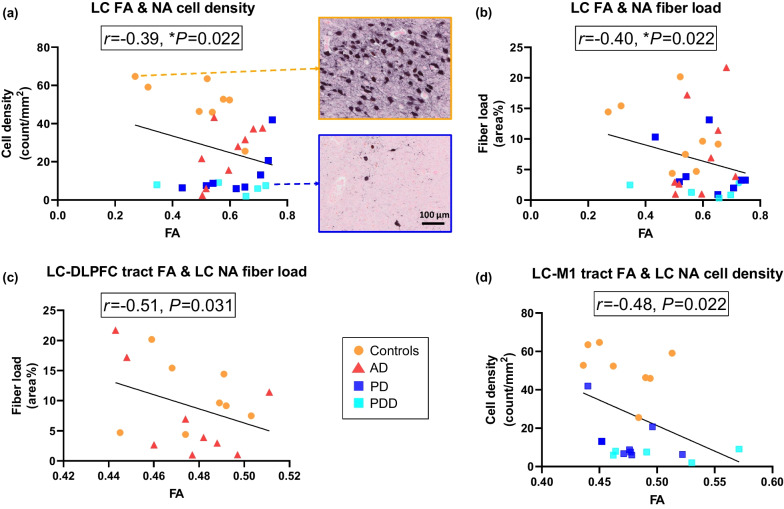
Microstructural alterations within the LC and LC-M1 tract correlate with LC-noradrenergic neuronal loss. **a** Increased FA of the LC was significantly correlated with reduced LC cell density in the whole cohort. Upper inserted image was from a control case showing low FA of the LC with high LC-noradrenergic cell density, lower image was from a PD case showing high FA of the LC with only a few LC-noradrenergic cells. Scale bar, 100 µm. **b** Increased LC FA was significantly correlated with reduced LC noradrenergic fiber load in the whole cohort. **c** Increased FA of the LC-DLPFC tract was significantly correlated (uncorrected) with reduced LC-noradrenergic fiber load in the LC of AD and control cases. **d** Increased FA of the LC-M1 tract was significantly correlated (uncorrected) with reduced noradrenergic cell density in the LC in PD and control cases

Within the whole cohort, we found that an increased FA of the LC was correlated with reduced noradrenergic cell density (*r* =  − 0.39, *P* = 0.022, FDR-corrected, Fig. 5a) and fiber load (*r* =  − 0.40, *P* = 0.022, FDR-corrected, Fig. 5b). In the PD group, increased FA of the LC was correlated with reduced noradrenergic fiber load (*r* =  − 0.64, *P* = 0.045, FDR-corrected), but not with cell density (*P* = 0.231). No correlations were found for the AD group (*P* = 0.148 and *P* = 0.304, for cell density and fiber load, respectively). No correlation was found between the MD of LC with noradrenergic cell density (*P* = 0.148) or fiber load (*P* = 0.304).

Although we did not find any differences in MRI tract measures between groups, microscopically we observed alterations in cortical DBH-immunoreactivity within the DLPFC and M1, and LC neuronal loss, suggesting that the fiber bundles may be affected. To this end, we explored the correlations between MRI measures of the LC-DLPFC tract and the LC-M1 tract with LC noradrenergic cell density and fiber load, as well as with cortical DBH^+^ load within the DLPFC and M1. For the LC-DLPFC tract, increased FA correlated significantly with reduced LC noradrenergic fiber load (*r* =  − 0.51, *P* = 0.031), and at the trend-level with reduced LC noradrenergic cell density (*r* =  − 0.48, *P* = 0.054) in the combined AD and control group, but not in the AD group alone. For the LC-M1 tract, increased FA significantly correlated with reduced LC noradrenergic cell density (*r* =  − 0.48, *P* = 0.022, Fig. 5C) in the combined PD and control group. Within the PD group only, an increased FA showed a trend of correlation with reduced LC noradrenergic cell density (*r* =  − 0.52, *P* = 0.063). No correlations were found between MD and LC noradrenergic cell and fiber loss, or between tract measures and cortical DBH^+^ load (Fig. 5, Additional file 1: Table S8).

In summary, microstructural alterations of the LC-DLPFC and the LC-M1 tracts may be driven by the LC noradrenergic cell loss, rather than by noradrenergic denervation of the cortex.

## Discussion

Using a combined MRI and histopathology approach, we investigated the sensitivity of diffusion MRI measures in detecting noradrenergic degeneration within the LC and its tracts in AD and PD. On diffusion MRI, an increased FA within the LC was found in the AD group, which was correlated with a loss of LC noradrenergic cells and fibers. In addition, increased FA of the LC-to-DLPFC tract was correlated with LC-noradrenergic fiber loss in the combined AD and control group, whereas the LC-to-M1 tract was correlated with LC noradrenergic neuronal loss in the combined PD and control group. The tract alterations were not correlated with cortical noradrenergic innervation or denervation. Altogether, our results suggest that the noradrenergic-related alterations within the LC and its tracts on diffusion MRI may be driven by the local noradrenergic neuronal loss within the LC, rather than by noradrenergic changes in the cortex.

In AD and PD, the LC undergoes severe noradrenergic neuronal loss, and aligning with previous evidence [30, 56, 67–71], we found significant loss of both noradrenergic neurons and fibers within the LC of AD and even more so in PD donors. The loss of noradrenergic neurons was not correlated with p-tau, Aβ or α-syn pathological hallmarks of AD or PD. While p-tau is suggested to be the main player in neuronal death in AD [72–74], the association may only be evident at early stage of the disease with less apparent neuronal loss but abundant p-tau pathology [70, 75]. In PD, the process of LB formation has been shown to associate with neurodegeneration [76–78]. In addition, both p-tau neurofibrillary tangles and α-syn LBs  are intracellular formations, and diminish along with cell death. Altogether, as shown in our study, both neuronal loss and pathological burden within the LC may reach a plateau at later stages of AD and PD.

We found no correlations between disease duration and noradrenergic cell density and fiber load in the LC of AD and PD donors. The results both align and contradict with previous literature [68, 79]. In the study by Zarow et al. [79], the PD disease duration was not correlated with neuronal loss in the LC but with the neuronal loss in the substantia nigra. The dopaminergic neuronal loss in the substantia nigra was shown to be associated with progression of motor severity throughout the disease [80, 81], whereas the LC is more involved at earlier stages of the disease in both cognitive and motor control. In AD, a positive correlation was found between disease duration and LC neuronal loss [68, 79], but this was not shown in our cohort, which may be due to the heterogeneity of our AD cohort, as the AD group consisted of EOAD and LOAD (Additional file 1: Fig. S6). EOAD is considered a devastating form of AD with fast deterioration of both clinical symptoms and brain pathology within a short disease duration [80, 82]. LC atrophy is greater in EOAD than in LOAD [83]. In our cohort, EOAD cases with disease duration < 7 years have an average 81% loss of LC neurons, which is comparable to the LOAD with disease duration > 10 years. In addition, EOAD cases with homozygous *APOE4* showed severe LC neuronal loss of 98% within only 2–6 years of disease duration (Additional file 1: Fig. S6) [84, 85]. However, this speculation was based on a descriptive observation in a very small sub-cohort of AD donors, and the interpretation requires caution [86, 87].

In our study, the FA of LC was higher in AD compared to controls, though it did not survive the correction for multiple comparisons. This may be due to the small sample size and the heterogeneity within the AD group, which consisted of EOAD and LOAD that showed differential noradrenergic degeneration when *APOE4* is taken into account. Currently, only two studies have examined LC integrity using diffusion MRI: in aging, increased FA was reported in the elderly compared to young adults [26]; in PD, higher FA was reported in patients with rapid-eye movement (REM) sleep disorder, compared to patients without this disorder, suggesting that the FA of LC is sensitive in detecting REM sleep disruptions induced by noradrenergic dysfunction at a prodromal stage of the disease. These studies indicate the capacity of FA to detect age- and disease-related alterations in the LC, as well as its connection to clinical symptoms. However, none of these studies provide a direct link towards the biological interpretation. Here, we showed that an increased FA may reflect pathological noradrenergic loss within the LC. Based on the theoretical diffusion model, the conventional interpretation of increased FA tends to be associated with increases of microstructural tissue elements, such as higher neuronal density, more intact membrane integrity and in turn less membrane permeability and greater myelination. This would be true in voxels with predominantly parallel running fibers, such as the corpus callosum. However, when the modeled FA is located at a crossing fiber region, the loss of crossing fibers would increase the FA (as a major loss in one of the crossing fiber bundles would turn the oblate tensor with a relatively low FA into a prolate tensor with a relatively high FA) in aging and neurodegeneration [25, 80, 88–91]. As the LC is located in the brainstem between the superior cerebellar peduncle, sensory nucleus trigemenal nerve, reticular formation and transverse pontine tracts, thus an environment of complex fiber architecture, loss of LC noradrenergic neurons and brainstem tracts could diminish the amount of crossing fibers [92, 93]. This would contribute to an elongated principal axis of water diffusion and thus increased FA. In contrast, MD would remain unchanged or increased with the loss of crossing fibers. We did not find group differences in the MD of LC, which may suggest that this measure is not sensitive enough in detecting noradrenergic degeneration. However, due to the small sample size and inclusion of late-stage disease donors, the results of the association of FA with neuronal loss in the LC should be interpreted with caution.

We did not find any tract difference at the group level but found correlations between tract measures and LC neuronal loss in the combined group. Previous studies have shown increased free water and diffusivity measures of the LC–transentorhinal tract in AD, which correlate with poor memory performance and increased AD pathology in the CSF [35, 94], suggesting that diffusion MRI has the potential to capture pathological changes at an early disease stage. In our study, although tract measures did not differentiate the diseased groups from the control group, they were associated with LC neuronal loss. The lack of group difference may be due to the aforementioned small sample size and heterogeneity within the groups.

Previous studies in PD have shown reduced noradrenergic transporters in the ACC, DLPFC and hippocampus compared to controls [7, 34]. In our study, the PD donors showed a trend of lower DBH^+^ load in these cortical regions (Fig. 4). Nonetheless, we found noradrenergic denervation in the motor cortex of PD donors compared to controls. This is consistent with previous post-mortem and in vivo imaging studies, which showed a loss of noradrenergic fibers and terminals and reduced ^11^C-MeNER binding in the M1 region, associated with clinical motor severity and disease progression [32, 95]. In our study, the M1 noradrenergic denervation was correlated with the LC-noradrenergic cell loss. Although this correlation was at a trend-level, this may imply axonal degeneration between the LC and the M1. Supporting evidence for this was the increased FA of the LC–M1 tract and its association with the LC- noradrenergic cell loss in the combined PD and control group. This result also suggests that the tract integrity is associated with noradrenergic changes in the seed region (LC) rather than the projecting region (M1). As our cohort consisted of late-stage disease donors with a substantial loss of LC-noradrenergic neurons, the change in diffusion MRI measures in our study may reflect this late event of LC noradrenergic degeneration. The loss of noradrenergic terminals in the LC-projecting regions has been observed before the occurrence of substantial neuronal loss within the LC, implying a retrograde mechanism [56, 96–100]. Further investigations in a larger cohort with different disease stages are needed to confirm this and assess the sensitivity of diffusion MRI for early LC-noradrenergic degeneration.

In AD, cell loss within the rostral LC is expected to affect hippocampal and prefrontal noradrenergic innervation [66]. Interestingly, here we found an increased DBH-immunoreactivity in the DLPFC of AD donors and no difference in the hippocampus. Previous post-mortem studies on AD and dementia with Lewy body disease found increased mRNA expression of tyrosine hydroxylase and noradrenergic synthesis in (remaining) LC neurons, accompanied by increased α_2_-adrenoreceptors and noradrenaline binding sites in the dendritic areas of the LC, hippocampus and PFC [101, 102]. This suggests noradrenergic dendritic and axonal sprouting to LC dendritic regions and projecting cortical regions. This process might be one of the compensatory mechanisms of the LC-noradrenergic system in AD [103, 104]. Results from our study may support this compensatory effect, as the sprouting of noradrenergic axon terminals, particularly in the DLPFC of AD donors, was observed (Additional file 1: Fig. S8). Unlike non-monoaminergic axons with only terminal boutons, the cortical-innervating noradrenergic axons possess beaded varicosities along the axon terminals that are in close contact to cortical interneurons and astrocytes [105]. Noradrenaline, together with densely expressed noradrenergic receptors in cortical interneurons, modulate the excitatory and inhibitory response and mediate prefrontal functioning, such as working memory and attention processing [106–110]. The axonal sprouting and increased noradrenergic immunoreactivity in the PFC of AD donors may then be the mending mechanisms for prefrontal functioning [111]. In fact, an increased CSF level of noradrenergic metabolite 3-methoxy-4-hydroxyphenylglycol (MHPG) has been found in AD patients at advanced stages of the disease [6, 112, 113], suggesting higher noradrenergic demands and turnover to support prefrontal functioning. This is an aberrant process rather than a beneficial process, as elevated MHPG levels have been associated with cognitive dysfunction as well as the spread and formation of p-tau and Aβ [114]. Moreover, accumulation of p-tau and Aβ, together with elevated MHPG levels, is associated with lower cortical thickness in LC-projecting regions [115]. These aberrant processes may suggest overactivation of the LC-noradrenergic circuitry for increased noradrenaline levels and a high demand on the system in neurodegenerative diseases. Nevertheless, further investigations are needed to confirm these speculations, which may aid in the development of therapeutic targets within the noradrenergic system [103].

Some limitations of this study should be addressed. First, although a sample size of 31 cases is considered large for in situ MRI-pathology studies, our results require replication in a larger cohort with donors from different stages of disease, to further validate diffusion MRI as a sensitive tool to detect noradrenergic degeneration in AD and PD. Second, although the AD and PD groups in our cohort were age- and sex-matched with controls, the inclusion of late disease stage cohort was not suitable for assessing early degeneration of the LC-noradrenergic system. In future studies, prodromal AD and preclinical PD cases are suggested to be included. The current study also shows the limitation of using LC diffusion metrics as an (early) biomarker for AD, as no group differences or associations with pathological hallmarks were found. We assessed noradrenergic degeneration in cortical regions that are highly affected in AD and PD, but the noradrenergic axons innervate throughout the neocortex, with dense innervation to the thalamus and dentate gyrus that supports storage of synaptic information and memory formation, which can be of interest for future studies [116]. Diffusion MRI can model and assess microstructure of white matter tracts, and its diffusion markers, FA and MD, are shown to be pathologically sensitive in neurodegenerative diseases [21, 25, 117–119]. However, the signal-to-noise ratio and the partial volume effects of nearby CSF, may affect the reproducibility of FA and MD in small regions such as the LC. Although we have validated the results by fitting the FA and the MD to the data in different shells, the results of the current study require further validation using diffusion MRI with higher spatial resolution. Given the fact that the tensor model is based on one shell, which is compromised in regions where fibers cross, multi-shell models such as kurtosis imaging and neurite orientation dispersion and density imaging can be used for future studies. In addition, the LC mask registration in T1 and DWI may lead to overestimation of the LC; in turn, the extracted ROI is likely to be LC-enriched instead of LC-specific, as the average LC volume in our study is higher than the volume described in previous stereological studies [56, 64] (Additional file 1: Table S2), although our estimation is shown to be closer to the estimation in stereological studies compared to previous MRI studies [120, 121]. As an alternative, LC-sensitive MRI has a better capacity to segment the LC and differentiate the rostral and caudal LC [12], which are differentially vulnerable in AD and PD [68]. Changes of LC signal intensity on LC-sensitive MRI have been shown to be a potential biomarker for monitoring disease progression and assessing behavioral improvement on noradrenergic treatments [12, 15, 19, 20, 122–124]. Future studies are encouraged to combine LC-sensitive MRI and diffusion MRI for LC segmentation and LC tractography.

## Conclusion

In conclusion, our study was the first to show that LC-noradrenergic neuronal loss, rather than cortical noradrenergic denervation, is associated with reduced integrity of the LC and its tracts, measured by diffusion MRI, in AD and PD. Combining LC-sensitive imaging with diffusion MRI would aid in the development of sensitive tools to detect changes in the LC-noradrenergic system in neurodegenerative diseases.

### Supplementary Information

**Additional file 1: Fig. S1.** LC 3D T1 and DWI space registration and transformation in an example control case. **Fig. S2.** LC delineation and pathology quantification. **Fig. S3.** DBH staining and Qupath quantification in the cortex. **Table S1.** Detail donor information. **Table S2.** LC volume in T1 and DWI space of controls, AD and PD donors. **Table S3.** FA and MD of LC in *b* = 2000 s/mm^2^ and *b* = 1000 s/mm^2^ shells, and group comparison results. **Table S4.** FA and MD (b0-b2000 shell) of LC tracts to ACC, DLPFC, M1 and hippocampus in controls, AD and PD, and the group comparison. **Fig. S4.** Pathological burden within the LC in AD, PD and controls. **Fig. S5.** An AD case with α-syn immunoreactivity in the LC. **Table S5.** Correlations between LC noradrenergic cell density and fiber load and pathological hallmarks. **Table S6.** Correlations between disease duration and LC noradrenergic cell density and fiber load in AD and PD. **Table S7.** Correlations between age and DBH+ whole cohort. **Fig. S6. **Correlation between disease duration and LC neuronal loss. **Fig. S7.** DBH staining pattern and noradrenergic fibers identified in ACC, D LPFC, M1 and hippocampus of controls, AD and PD. **Fig. S8.** Axonal sprouting in the DLPFC of two AD cases. **Fig. S9.** DBH staining validation with Novus (Campbridge, UK). **Table S8.** Correlations between FA and MD of the LC-DLPFC and LC-M1 tracts with LC noradrenergic cell density and fiber load. Supplementary materials for the script of LC meta mask registration using Advanced Normalization Tools (ANTs, version 2.1).

## Data Availability

The datasets generated during and/or analyzed during the current study are available from the corresponding author on reasonable request. Supporting materials include supplementary methods, tables and figures.

## Abbreviations

LC-noradrenergic: Locus coeruleus-noradrenergic
AD: Alzheimer’s disease
PD: Parkinson’s disease
PDD: Parkinson’s disease with dementia
FA: Fractional anisotropy
MD: Mean diffusivity
ACC: Anterior cingulate cortex
DLPFC: Dorsolateral prefrontal cortex
M1: Primary motor cortex
DBH: Dopamine-beta hydroxylase
DWI: Diffusion weighted imaging
p-tau: Phosphorylated-tau
Aβ: Amyloid-beta
LB: Lewy body
LN: Lewy neurite
NABCA: Normal Aging Brain Collection Amsterdam
