# Supplementary material for: Microstructural integrity of the locus coeruleus and its tracts reflect noradrenergic degeneration in Alzheimer’s disease and Parkinson’s disease
Source: Transl Neurodegener. 2024 Feb 9;13:9. doi: 10.1186/s40035-024-00400-5 (PMC10854137; doi:10.1186/s40035-024-00400-5)
Supplement: Supplementary file 1 — Additional file 1: Fig. S1. LC 3D T1 and DWI space registration and transformation in an example control case. Fig. S2. LC delineation and pathology quantification. Fig. S3. DBH staining and Qupath quantification in the cortex. Table S1. Detail donor information. Table S2. LC volume in T1 and DWI space of controls, AD and PD donors. Table S3. FA and MD of LC in b = 2000 s/mm2 and b = 1000 s/mm2 shells, and group comparison results. Table S4. FA and MD (b0-b2000 shell) of LC tracts to ACC, DLPFC, M1 and hippocampus in controls, AD and PD, and the group comparison. Fig. S4. Pathological burden within the LC in AD, PD and controls. Fig. S5. An AD case with α-syn immunoreactivity in the LC. Table S5. Correlations between LC noradrenergic cell density and fiber load and pathological hallmarks. Table S6. Correlations between disease duration and LC noradrenergic cell density and fiber load in AD and PD. Table S7. Correlations between age and DBH+ whole cohort. Fig. S6. Correlation between disease duration and LC neuronal loss. Fig. S7. DBH staining pattern and noradrenergic fibers identified in ACC, D LPFC, M1 and hippocampus of controls, AD and PD. Fig. S8. Axonal sprouting in the DLPFC of two AD cases. Fig. S9. DBH staining validation with Novus (Campbridge, UK). Table S8. Correlations between FA and MD of the LC-DLPFC and LC-M1 tracts with LC noradrenergic cell density and fiber load. Supplementary materials for the script of LC meta mask registration using Advanced Normalization Tools (ANTs, version 2.1). [file 40035_2024_400_MOESM1_ESM.docx]

**Supplementary Materials**

**Methods**

**Immunohistochemistry for dopamine-beta hydroxylase (DBH), phosphorylated-alpha-synuclein (pSer129- αsyn), phosphorylated-tau (p-tau) and amyloid-beta (Aβ)**

All tissue blocks were formalin-fixated and paraffin-embedded (FFPE), cut with a Microtome (Leica, Germany), and mounted onto superfrost plus glass slides (Thermo Scientific, USA) for subsequent immunohistochemistry. The LC blocks were cut at 4 x 20 µm serial sections for four single stainings: rabbit-anti DBH (dilution 1:400, Abcam, Cambridge, UK), rabbit-anti pSer129-asyn (clone EP1536Y, dilution 1:4000, Abcam, Cambridge, UK), mouse-anti p-tau (clone AT8, dilution 1:800, ThermoFisher, Pittsburgh, USA) and mouse-anti Aβ (clone 4G8, dilution 1:5000, BioLegend, San Diego, USA). The cortical blocks, namely the anterior cingulate cortex (ACC), dorsolateral prefrontal cortex (DLPFC), precentral gyrus (M1) and hippocampus, were cut at 6 µm for one single staining: rabbit-anti DBH (dilution 1:400, Abcam, Cambridge, UK).

For immunohistochemistry, all sections were deparaffinized and dehydrated in xylene and a graded series of ethanol. After this, sections underwent antigen retrieval in citrate buffer (pH 6.0) at a temperature of 95°C. Thereafter, sections were first blocked for endogenous peroxidase by immersing the sections in 1% of H_2_O_2_ in tris-buffered saline (TBS, pH 7.4) for 30 minutes, and subsequently blocked with 3% normal goat serum in TBS with 0.1% Triton x-100. LC sections were incubated with primary antibodies of either DBH, pSer129-**α**syn, p-tau or Aβ, whereas the sections of cortical regions were incubated with primary DBH antibody of DBH. The incubated primary antibodies were diluted in 3% normal goat serum in TBS + 0.1% Triton x-100 for two nights at 4°C, followed by Immpress-HRP anti-rabbit (for DBH and pSer129-asyn) or anti-mouse (for p-tau and Aβ) detection (Vector, California, United State). Finally, DBH was visualized with 3,3′-diaminovenzidine (DAB, Sigma-Aldrich, Germany) with nickel. pSer129-**α**syn, p-tau and Aβ were visualized with Vector SG (Vector, California, United State) and counterstained with nuclear fast red (Vector, California, United State). Lastly, sections were dehydrated in a graded series of ethanol, xylene and mounting with Entellan.

**Immunohistochemistry for DBH validation**

One representative sections from each AD, PD and control group, was selected for validating the above mentioned DBH staining from Abcam (Cambridge, UK), using Novus (Cambridge, UK, dilution 1:100). One DLPFC section at 6 µm per case went through the same deparaffinization and rehydration steps as previously mentioned. After antigen retrieval in citrate buffer (pH 6.0), the sections were washed for two nights. Thereafter, sections were blocked using the same method and incubated with primary antibody of DBH (Novus) diluted in 3% normal goat serum in TBS + 0.1% Triton x-100, for two nights. Secondary antibody and visualization were the same as the procedure for Abcam DBH mentioned above.

**Quantification of DBH, pSer129-α-Syn, p-tau and Aβ immunoreactivity in the LC**

To quantify DBH+ cell bodies, an in-house script was developed that first identified and then counted all cells within a 2.5mm^2^ sampling grid and subsequently selected only the ones that were positive to DBH. In a next step, cell bodies were subtracted from the annotation, and the script determined the %area load of DBH+ threads with a pixel classifier. Lewy bodies were detected using a cell detection based on optical density sum, specified with the following classifiers: a 5-25 µm (or an area between 28.27 and 490.9 µm^2^ ). Subsequently, Lewy bodies were extracted from the ROI to further quantify Lewy neurites using a pixel classifier. P-tau and Aβ were quantified using pixel classifiers into total p-tau and Aβ load expressed as area% immunoreactivity (Supplementary Fig. 1).


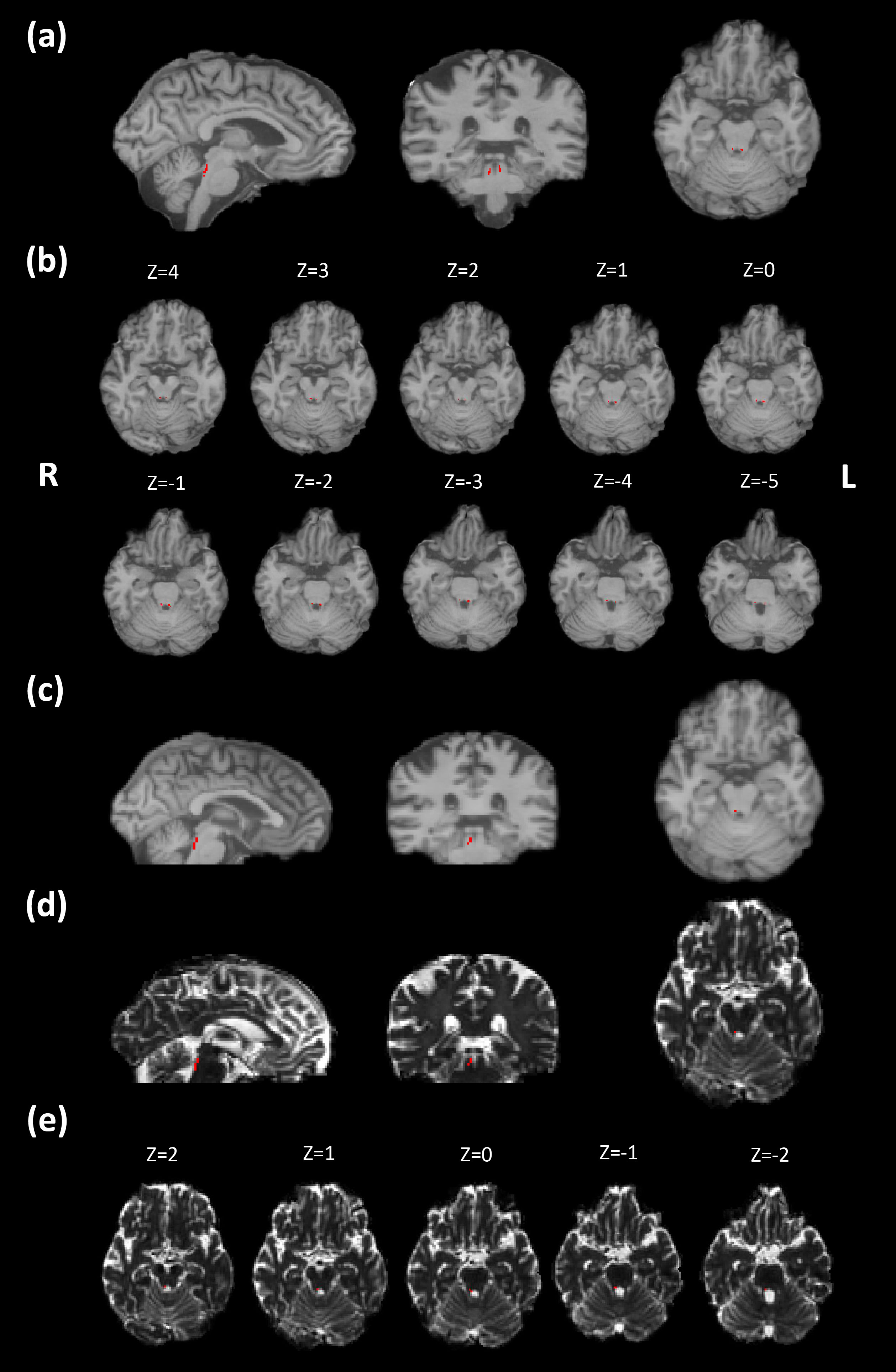


**Fig. S1. LC 3D T1 and DWI space registration and transformation in an example control case.** (**a**) a LC meta mask (in red) was first registered to T1 space using Advanced Normalization Tools (version 2.1; ANTs), and shown in overlay with the brain in sagittal, coronal and axial view in T1 space. (**b**) The LC mask overlay with the brain in axial view, showing 5 slices of 2 mm, covering 10 mm in superior-inferior direction. (**c**) The LC mask was then transformed from T1 to DWI using linear transformation. To ensure the transformation between T1 and DWI is valid, we check the co-registration of the brain from T1 to DWI, as well as the LC mask in DWI space. Only the LC mask of the right hemisphere was used for registration in DWI. (**d**) The LC mask of the right hemisphere overlay on DWI (b=0) in sagittal, coronal and axial view. (**e**) The LC mask overlay with the brain in axial view, showing 10 slices of 1 mm, covering 10 mm in superior-inferior direction. The brain images are shown in radiological view (R-L). The registration script for LC meta mask with detail registration parameters Advanced Normalization Tools (ANTs, version 2.1) is at the end of the Supplementary materials.


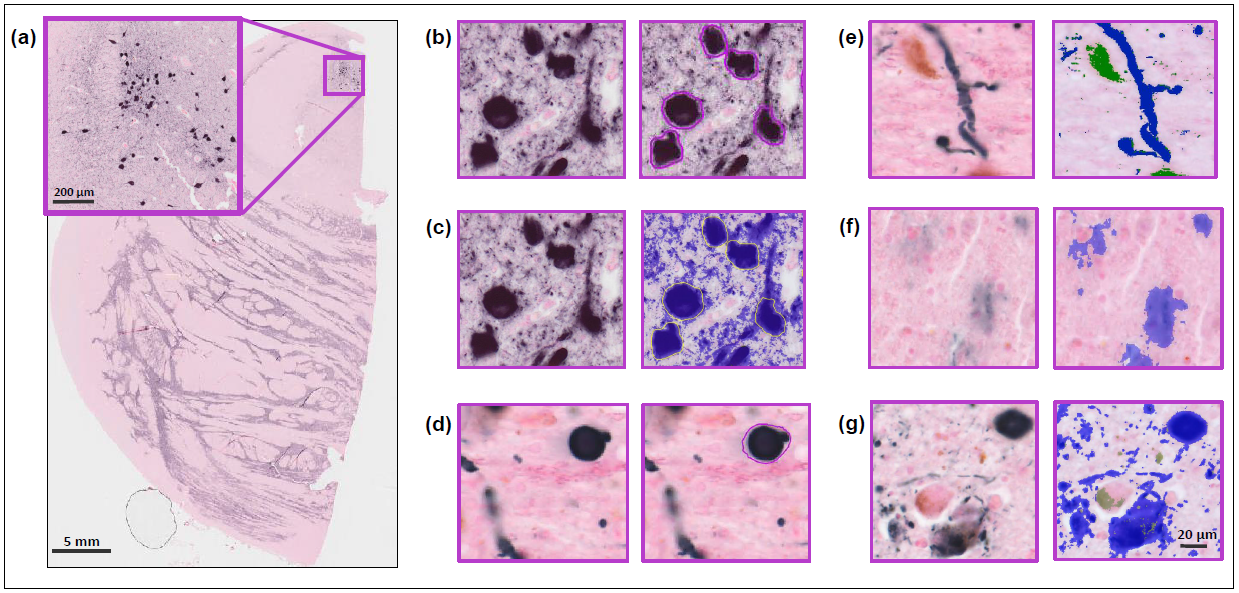


**Fig. S2. LC delineation and pathology quantification.** Based on the delineation method developed from stereology (1-3), the LC was defined by placing a 2.5mm^2^ sampling grid at the center of a cluster of neuromelanin-containing cells on the DBH-stained sections. With the aid of neighboring anatomical landmarks, namely the fourth ventricle, mesencephalic trigeminal nucleus and its tract. The sampling grid was placed at a similar level of the LC in each case. Digital quantification of (**b**) LC noradrenergic neurons, (**c**) LC noradrenergic fibers, (**d**) a Lewy body, (**c**) a Lewy neurite, (**f**) Aβ and (**g**) p-tau with in-house developed scripts. The scale bar of 20 µm applies to all images of **b-g**.


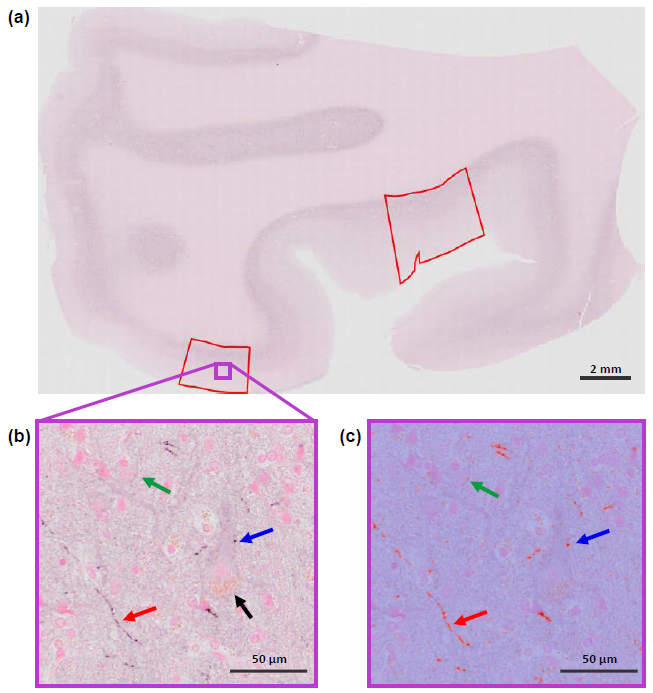
**Fig. S3. DBH staining and Qupath quantification in the cortex.** (**a**) The DBH staining of the primary motor cortex section from a control case. The regions of interest containing all cortical layers were delineated in straight areas of the cortex, as shown in red delineations. (**b**) The zoomed-in illustration of the staining in one of the ROIs showed a Betz cell (black arrow), elongated DBH+ fiber (red arrow), dot-like cross-section fiber (blue arrow) and punctate staining that represent synaptic DBH (green arrow). (**c**) The aforementioned structures (elongated and dot-like fibers and synaptic DBH) were picked-up by Qupath scripts. We thus took the outcome of DBH quantification as combined DBH+ load (%).

**Results**

**Table S1. Detail donor information**

| Case | Group | Age at death (y) | Sex | PMD | Cause of death | Disease duration | Braak NFT stage | Thal Aβ phase | Braak LB | ABC | CAA | CDR | ApoE status |
| --- | --- | --- | --- | --- | --- | --- | --- | --- | --- | --- | --- | --- | --- |
| 1 | Control | 63 | F | 8:10 | Euthanasia | NA | 0 | 0 | 0 | A0 B0 C0 | 0 | NA | NA |
| 2 | Control | 85 | M | 9:20 | Euthanasia | NA | 1 | 1 | 0 | A1 B1 C0 | 0 | NA | NA |
| 3 | Control | 67 | M | 8:25 | Euthanasia | NA | 2 | 1 | 0 | A1B1C0 | 0 | NA | NA |
| 4 | Control | 67 | M | 8:35 | Liver cirrhosis | NA | 1 | 1 | 0 | A1 B1 C0 | 2 | NA | NA |
| 5 | Control | 57 | M | 9:50 | Euthanasia | NA | 0 | 1 | 0 | A1 B0 C0 | 0 | NA | NA |
| 6 | Control | 83 | F | 5:20 | Euthanasia | NA | 3 | 4 | 0 | A2 B2 C0 | 0 | NA | NA |
| 7 | Control | 84 | F | 5:00 | Euthanasia | NA | 3 | 1 | 0 | A1 B2 C0 | 2 | NA | NA |
| 8 | Control | 77 | M | 3:50 | Euthanasia | NA | 3 | 1 | 0 | A1 B2 C0 | 2 | NA | NA |
| 9 | AD | 53 | M | 9:00 | Palliative sedation | 5 | 6 | 5 | 0 | A3 B3 C3 | 1 | 3 | 33 |
| 10 | AD | 64 | M | 7:55 | Cachexia and dehyrdation | 12 | 6 | 5 | Amygdala only | A3 B3 C3 | 2 | 3 | 34 |
| 11 | AD | 84 | M | 8:35 | Euthanasia | 14 | 4 | 3 | 0 | A2 B2 C2 | 0 | NA | 33 |
| 12 | AD | 77 | M | 9:05 | Suicide | 10 | 6 | 5 | 0 | A3 B3 C3 | 1 | NA | 44 |
| 13 | AD | 65 | M | 9:20 | Euthanasia | 7 | 5 | 5 | 0 | A3 B3 C3 | 1 | 1 | 34 |
| 14 | AD | 63 | M | 8:45 | Myocardial infarction | 10 | 6 | 5 | 0 | A3 B3 C3 | 0 | 2 | 43 |
| 15 | AD | 61 | F | 7:40 | End-stage AD | 6 | 6 | 5 | 0 | A3 B3 C3 | 1 | NA | 44 |
| 16 | AD | 53 | F | 6:30 | End-stage AD | 7 | 6 | 5 | 0 | A3 B3 C3 | 1 | 2 | 23 |
| 17 | AD | 60 | M | 7:05 | Euthanasia | 3 | 6 | 5 | 0 | A3 B3 C3 | 1 | 1 | 44 |
| 18 | PDD | 74 | F | 8:10 | Euthanasia | 12 | 2 | 3 | 6 | A2B1C0 | 2 | 1 | NA |
| 19 | PD | 69 | F | 7:05 | Aspiration pneumonia | 15 | 2 | 2 | 6 | A1B1C0 | 0 | 0.5 | NA |
| 20 | PD | 82 | F | 9:17 | Aspiration pneumonia | 17 | 2 | 2 | 6 | A1B1C0 | 0 | NA | NA |
| 21 | PD | 78 | M | 7:15 | Euthanasia | 17 | 2 | 1 | 6 | A1B1C0 | 0 | 0.5 | NA |
| 22 | PDD | 62 | M | 8:15 | End-stage Parkinson's disease | 18 | 2 | 1 | 6 | A1B1C0 | 0 | NA | NA |
| 23 | PD | 75 | M | 4:30 | End-stage Parkinson's disease | 20 | 2 | 3 | 6 | A2B1C0 | 0 | 0.5 | NA |
| 24 | PD | 78 | M | 3:30 | End-stage Parkinson's disease | 20 | 2 | 1 | 6 | A1B1C0 | 0 | NA | NA |
| 25 | PDD | 74 | M | 8:50 | Aspiration pneumonia | 8 | 3 | 2 | 6 | A1B2C0 | 0 | 2 | NA |
| 26 | PDD | 81 | F | 5:30 | End-stage Parkinson's dementia | 19 | 2 | 3 | 6 | A2B1C0 | 0 | NA | NA |
| 27 | PDD | 70 | M | 6:55 | Euthanasia | 8 | 1 | 1 | 6 | A1B1C0 | 0 | 1 | NA |
| 28 | PD | 83 | M | 6:30 | Euthanasia | NA | 3 | 1 | 6 | A1B2C0 | 0 | NA | NA |
| 29 | PDD | 84 | M | 10:30 | Euthanasia | NA | 3 | 1 | 6 | A1B2C0 | 2 | NA | NA |
| 30 | PD | 85 | M | 7:10 | End-stage Parkinson's disease | 13 | 2 | 1 | 5 | A1B1C0 | 0 | 0.5 | NA |
| 31 | PD | 87 | M | 6:10 | Euthanasia | 19 | 2 | 3 | 6 | A2B1C0 | 0 | 0.5 | NA |

Abbreviations: AD, Alzheimer’s disease; PD, Parkinson’s disease; PDD, Parkinson’s with dementia; M, male; F, female; PMD, post-mortem delay; hr, hour; min, minutes; LB, Lewy body; NFT, neurofibrillary tangle; y,years; CAA, cerebral amyloid angiopathy. CDR, clinical dementia rating scale. ApoE, apolipoprotein. NA, not applicable or available.

**Table S2. LC volume in T1 and DWI space of controls, AD and PD donors.**

| Group | Average voxels in T1 (mm^3^) | Average volumes in T1 (mm^3^) | SD of volumes in T1 (mm^3^) | Average volumes in DWI (mm^3^) | Average volumes in DWI (mm^3^) | SD of volumes in DWI (mm^3^) |
| --- | --- | --- | --- | --- | --- | --- |
| Controls | 85.88 | 21.47 | 5.48 | 3.75 | 22.97 | 11.22 |
| AD | 85.33 | 21.33 | 5.20 | 3.89 | 23.82 | 8.90 |
| PD | 79.36 | 19.84 | 6.71 | 3.64 | 22.31 | 10.36 |

Abbreviations: AD, Alzheimer’s disease; PD, Parkinson’s disease; DWI, diffusion weighted imaging; SD, standard deviation.

**Table S3. FA and MD of LC in b=2000** **s/mm^2^ and b=1000 s/mm^2^ shells, and group comparison results.**

|  |  | **Controls** | |  | **AD** | |  | **PD** | |  | Group comparison |
| --- | --- | --- | --- | --- | --- | --- | --- | --- | --- | --- | --- |
|  |  | Mean | SD |  | Mean | SD |  | Mean | SD |  |  |
| **b=2000 s/mm^2^** | | | | | | | | | | | |
| FA |  | 0.496 | 0.135 |  | 0.625 | 0.105 |  | 0.586 | 0.106 |  | AD Vs. controls, #*p*=0.04 |
| MD (10^-3^ mm^2^ /s) |  | 0.507 | 0.167 |  | 0.406 | 0.085 |  | 0.454 | 0.139 |  | ns. |
| **b=1000 s/mm^2^** | | | | | | | | | | | |
| FA |  | 0.543 | 0.164 |  | 0.692 | 0.139 |  | 0.637 | 0.137 |  | AD Vs. controls, *p*=0.06 |
| MD (10^-3^ mm^2^ /s) |  | 0.623 | 0.252 |  | 0.481 | 0.134 |  | 0.547 | 0.210 |  | ns. |

Abbreviations: AD, Alzheimer’s disease; PD, Parkinson’s disease; FA, fractional anisotropy; MD, mean diffusivity; SD, standard deviation; ns, not significant. #*p*<0.05, uncorrected.

**Table S4. FA and MD (b0-b2000 shell) of LC tracts to ACC, DLPFC, M1 and hippocampus in controls, AD and PD, and the group comparison.**

| Tract FA |  | Controls | |  | AD | |  | PD | | Group comparison | |
| --- | --- | --- | --- | --- | --- | --- | --- | --- | --- | --- | --- |
|  |  | Mean | SD |  | Mean | SD |  | Mean | SD |  |  |
| ACC |  | 0.568 | 0.036 |  | 0.567 | 0.026 |  | 0.571 | 0.027 |  | ns. |
| DLPFC |  | 0.549 | 0.032 |  | 0.551 | 0.035 |  | 0.559 | 0.024 |  | ns. |
| M1 |  | 0.581 | 0.026 |  | 0.592 | 0.039 |  | 0.599 | 0.023 |  | ns. |
| Hippocampus |  | 0.547 | 0.035 |  | 0.551 | 0.058 |  | 0.555 | 0.040 |  | ns. |
|  |  |  |  |  |  |  |  |  |  |  |  |
| Tract MD  (10^-3^ mm^2^ /s) |  | Controls | |  | AD | |  | PD | | Group comparison | |
|  |  | Mean | SD |  | Mean | SD |  | Mean | SD |  |  |
| ACC |  | 0.357 | 0.041 |  | 0.355 | 0.029 |  | 0.361 | 0.029 |  | ns. |
| DLPFC |  | 0.353 | 0.039 |  | 0.344 | 0.027 |  | 0.350 | 0.023 |  | ns. |
| M1 |  | 0.364 | 0.039 |  | 0.354 | 0.028 |  | 0.364 | 0.033 |  | ns. |
| Hippocampus |  | 0.410 | 0.080 |  | 0.426 | 0.075 |  | 0.427 | 0.061 |  | ns. |

Abbreviations: AD, Alzheimer’s disease; PD, Parkinson’s disease; FA, fractional anisotropy; MD, mean diffusivity; ACC, anterior cingulate cortex; DLPFC, dorsolateral prefrontal cortex; M1, primary motor cortex; SD, standard deviation; ns, not significant.


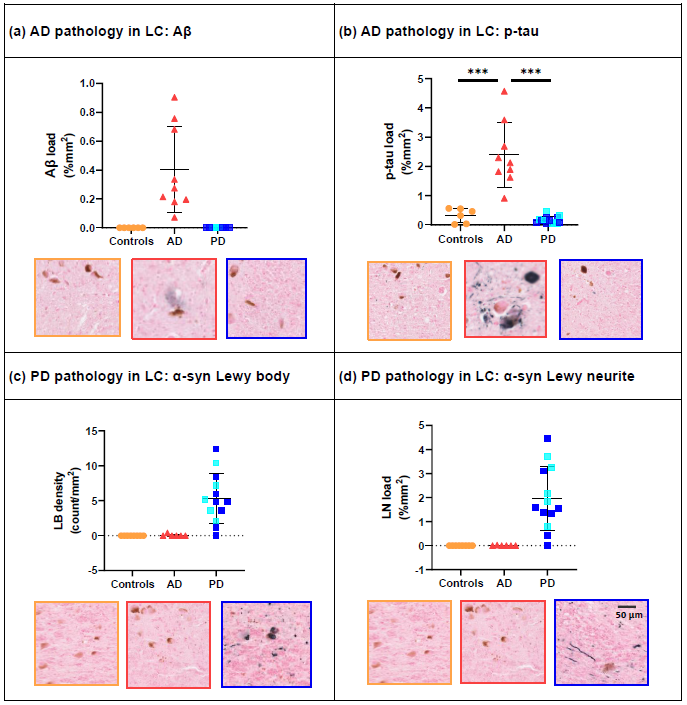
**Fig. S4. Pathological burden within the LC in AD, PD and controls.** (**a**) AD showed Aβ plaques, whereas PD and control cases did not. (**b**) compared to controls and PD cases, AD cases showed significantly higher p-tau load in the form of neurofibrillary tangles and threads. (**c, d**) PD cases showed accumulation of Lewy bodies and Lewy neurites, while AD and control cases did not. The scale bar of 50 µm applies to all the inserted representative images of each group. Within the PD group, PD is labeled with darker blue, whereas PDD is labeled with lighter blue. Abbreviations: AD, Alzheimer’s disease; PD, Parkinson’s disease; LB, Lewy bodies; LN, Lewy neurites; p-tau, phosphorylated-tau; Aβ, amyloid-beta; ns, not significant. ****p*<0.001 corrected.


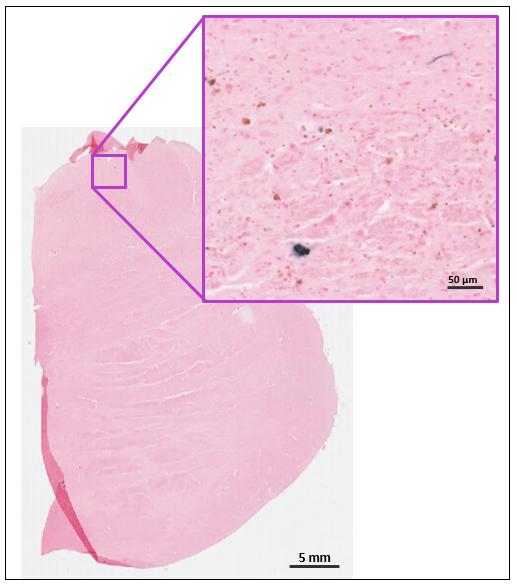
 **Fig. S5. An AD case with α-syn immunoreactivity in the LC.** We found an AD case with one LB in the LC. However, we did not find α-syn pathology in the dorsal motor nucleus of vagal nerve, therefore this case did not meet the criteria of Braak LB stage 1 (4), and is considered to have co-morbid α-syn pathology in the LC.

**Table S5. Correlations between LC noradrenergic cell density and fiber load and pathological hallmarks.**

|  |  | **LC NA cell density (count/mm2)** | |  | **LC NA fiber load (%)** | |
| --- | --- | --- | --- | --- | --- | --- |
|  |  | Pearson's r | p-value |  | Pearson's r | p-value |
| **AD** | **p-tau load (%)** | -0.140 | 0.411 |  | 0.599 | 0.143 |
|  | **Aβ load (%)** | -0.393 | 0.256 |  | -0.038 | 0.476 |
| **PD** | **LB density (count/mm^2^)** | 0.596 | 0.090 |  | 0.374 | 0.161 |
|  | **LN load (%)** | 0.441 | 0.117 |  | 0.355 | 0.175 |

Abbreviations: LC, locus coeruleus; NA, noradrenergic; AD, Alzheimer’s disease; PD, Parkinson’s disease; p-tau, phosphorylated-tau; Aβ, amyloid-β; LB, Lewy body; LN, Lewy neurite. **p*<0.05, corrected.

**Table S6. Correlations between disease duration and LC noradrenergic cell density and fiber load in AD and PD.**

|  | **LC NA cell density (count/mm2)** | | **LC NA fiber load (%)** | |
| --- | --- | --- | --- | --- |
|  | Pearson's r | p-value | Pearson's r | p-value |
| **AD** | | | | |
| **Disease duration (years)** | 0.294 | 0.286 | 0.217 | 0.340 |
| **PD** | | | | |
| **Disease duration (years)** | 0.255 | 0.271 | -0.330 | 0.212 |

Abbreviations: LC, locus coeruleus; NA, noradrenergic; AD, Alzheimer’s disease; PD, Parkinson’s disease. The percentage of neuronal loss was calculated with controls as baseline

**Table S7. Correlations between age and DBH+ whole cohort.**

| Region | Correlation with age | |
| --- | --- | --- |
|  | Pearson's r | p-value |
| LC NA cell density | -0.025 | 0.460 |
| LC NA fiber load | -0.053 | 0.415 |
| ACC | -0.139 | 0.259 |
| DLPFC | -0.298 | 0.058 |
| M1 | -0.189 | 0.172 |
| Hippocampus | -0.152 | 0.211 |

Abbreviations: ACC, anterior cingulate cortex; DLPFC, dorsolateral prefrontal cortex; M1, primary motor cortex.


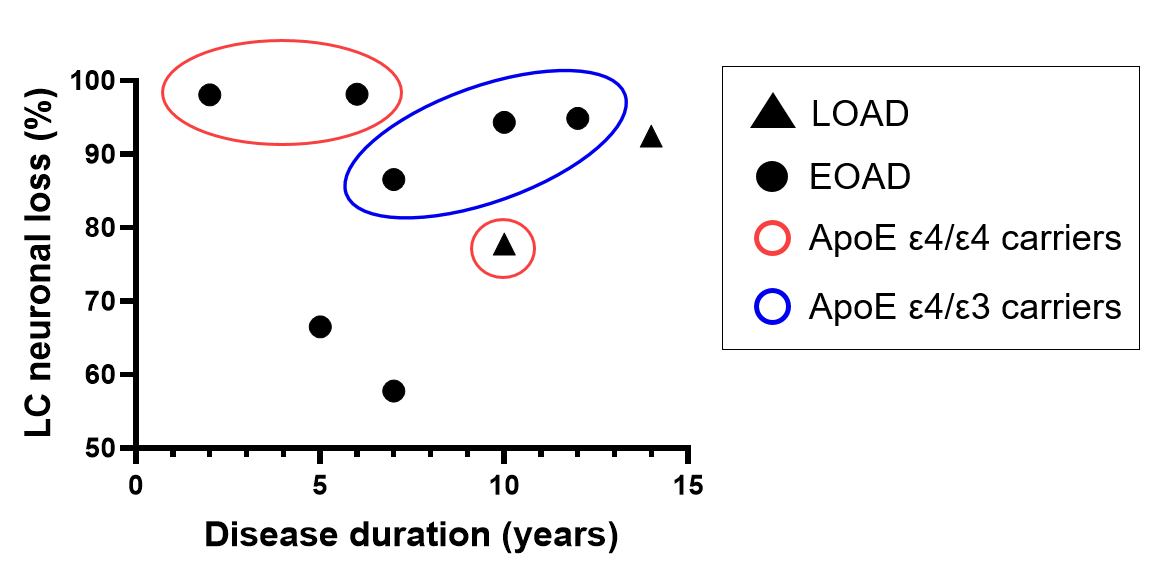


**Fig. S6.** Correlation between disease duration and LC neuronal loss. No correlation was found between disease duration and neuronal loss in AD cases (p=0.286, Supplementary Table 5). The AD cases consist of early- and late- onset AD case (EOAD and LOAD, respectively). EOAD with homozygous ApoE ε4 showed severe neuronal loss in the LC within a short disease duration. The percentage of neuronal loss was calculated with controls as baseline.


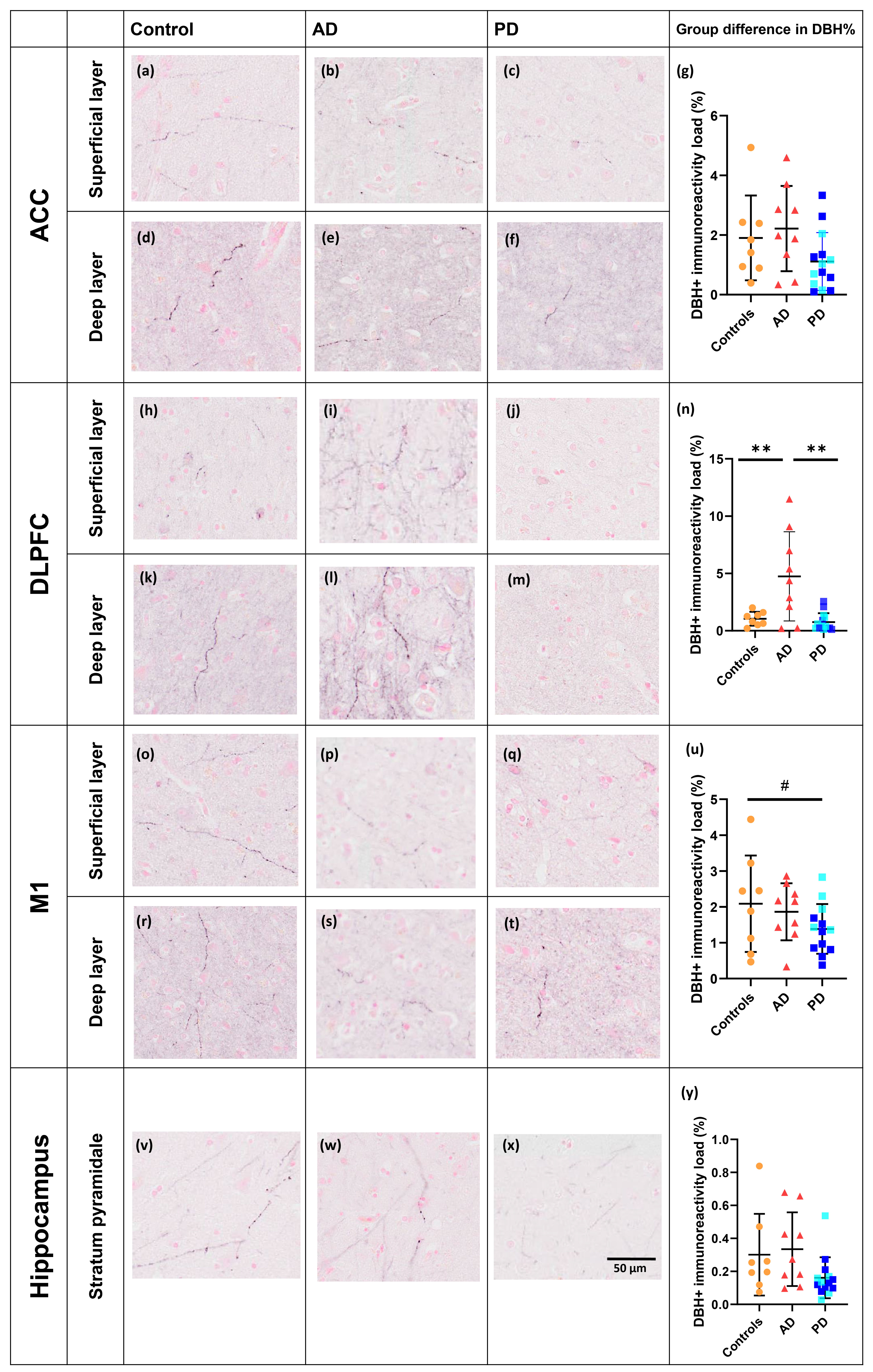


**Fig. S7. DBH staining pattern and noradrenergic fibers identified in ACC, DLPFC, M1 and hippocampus of controls, AD and PD.** The DBH staining pattern of the ACC superficial layers (**a-c**) show less punctate staining (synaptic DBH) than the deep layer (**d-f**). Noradrenergic fibers are observed with stronger signal intensity of DBH and elongated structures, and are descriptively more present in control **(a,d**) and AD **(b, e)** compared to PD (**c, f**) which is quantified in **(g**). The DBH staining pattern in the DLPFC superficial layers (**h-j**) show less synaptic DBH than the deep layers (K-M).Higher synaptic DBH and more noradrenergic fibers are identified in AD, for both superficial **(i**) and deep layer **(l**), compared to controls (**h, k**) and PD (**j, m**). This observation was also statistically significant (**n**). The DBH staining pattern in the M1 superficial layers (**o**-**q**) show less synaptic DBH than the deep layer (**r-t**) Controls show higher synaptic DBH and noradrenergic fibers (**o, r**) compared to PD (P, S) by both observation and statistical comparison (**u**). The DBH staining pattern in the pyramidale layer of hippocampus (**v-x**): the amount of noradrenergic fibers identified is higher in controls (**v**) and AD (**w**) than PD (**x**), though no significant group difference were found (**y**). The scale bar of 50 μm applies to all staining images in this figure. Within the PD group, PD is labeled with darker blue, whereas PDD is labeled with lighter blue.


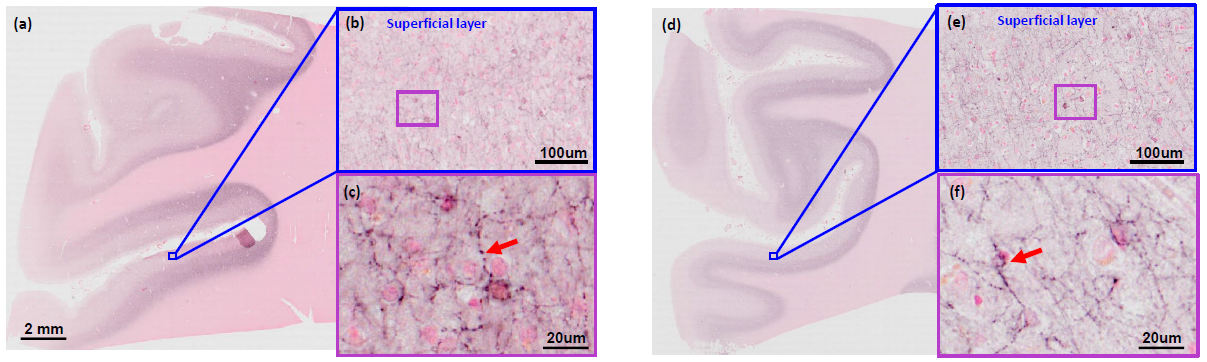


**Fig. S8. Axonal sprouting in the DLPFC of two AD cases.** (**a**) The DLPFC stained with DBH of a 64 year-old AD case with a disease duration of 12 years. (**b**) Zoom-in of the superficial layers (scale bar 100 µm) showing many noradrenergic fibers and synaptic DBH. (**c**) Zoom-in of the purple square in (**b**) at 20 µm, showing the noradrenergic fibers with beading varicosities surrounding the soma of cortical neurons (red arrow). (**d**) The DLPFC section stained with DBH of a 77 year-old AD case with a disease duration of 10 years. (**e**) Zoom-in of the superficial layer at 100 µm showing many noradrenergic fibers and synaptic DBH. (**f**) Zoom-in of the purple square in (**e**) at 20 µm, showing another noradrenergic fiber with varicosities traveling on top of a cortical neuron (red arrow).


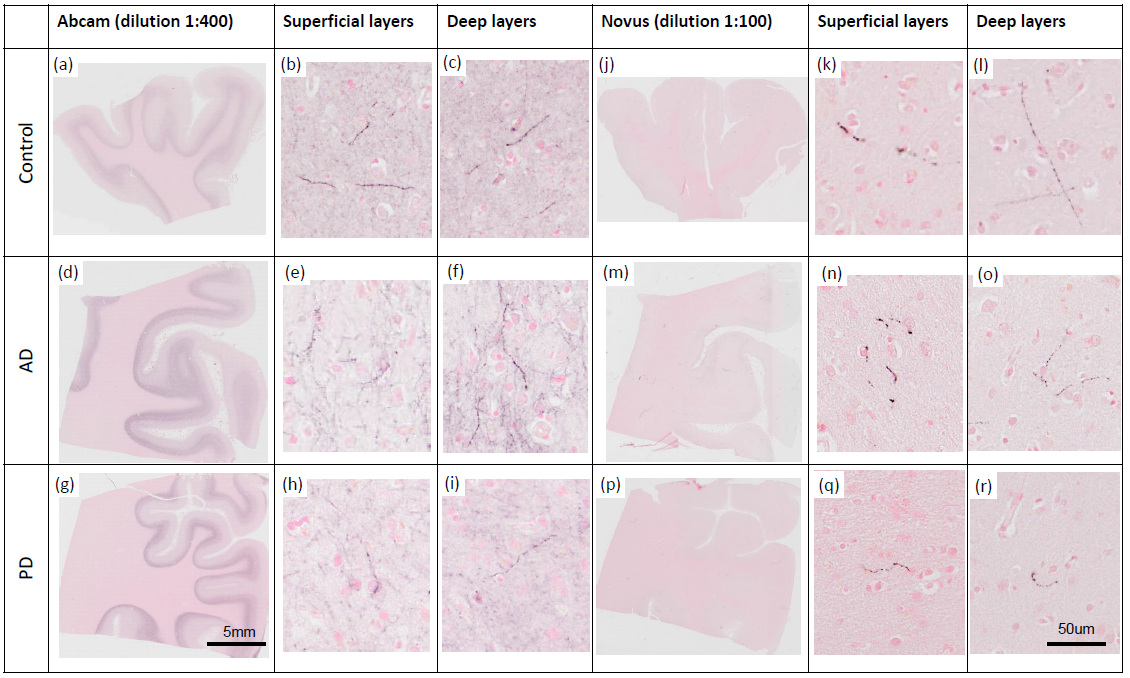
 **Fig. S9. DBH staining validation with Novus (Campbridge, UK). a** DLPFC section of a control, AD and PD case was stained for Rabbit-anti DBH (dilution 1:100, Novus, Cambridge, UK) to validate the DBH stainings of Abcam (dilution 1:400, Abcam, Cambridge, UK), which was used in the main study. Between the two antibodies, the Abcam antibody (**a-i**) showed higher synaptic DBH and more noradrenergic fibers compared to Novus antibody **(j-r)**. However, both antibodies showed similar morphological patterns for the noradrenergic fibers, elongated fibers with stronger signal intensity. AD shows more noradrenergic fibers (**e,f** and **n,o**), and they travel around the soma of cortical neurons, compared to control (**b,c** and **k,l**) and PD (**h,i** and **q, r**) . The scale bar of 5mm applies to the section images, A,D,G,J,M,P; the scale bar of 50 μm applies to the zoom-in images of sections, **b, c, e, f, h, i, k, l, n, o, q, r**.

**Table S8. Correlations between FA and MD of the LC-DLPFC and the LC-M1 tracts with LC noradrenergic cell density and fiber load.**

|  |  |  |  |  |  |
| --- | --- | --- | --- | --- | --- |
| **AD and controls** | **DLPFC tract FA** | |  | **DLPFC tract MD (10^-3^ mm^2^/s)** | |
|  | Pearson's r | p-value |  | Pearson's r | p-value |
| **LC NA cell density (count/mm^2^)** | **-0.447** | **0.054** |  | -0.169 | 0.282 |
| **LC NA fiber load (%)** | **-0.510** | **0.031** |  | -0.121 | 0.340 |
| **DLPFC DBH+ load (%)** | -0.022 | 0.470 |  | 0.137 | 0.320 |
|  |  |  |  |  |  |
| **AD** | **DLPFC tract FA** | |  | **DLPFC tract MD (10^-3^ mm^2^/s)** | |
|  | Pearson's r | p-value |  | Pearson's r | p-value |
| **LC NA cell density (count/mm^2^)** | -0.414 | 0.207 |  | -0.390 | 0.222 |
| **LC NA fiber load (%)** | -0.406 | 0.212 |  | **-0.634** | **0.088** |
| **DLPFC DBH+ load (%)** | -0.117 | 0.413 |  | -0.390 | 0.222 |
|  |  |  |  |  |  |
| **PD and controls** | **M1 tract FA** | |  | **M1 tract MD (10^-3^ mm^2^/s)** | |
|  | Pearson's r | p-value |  | Pearson's r | p-value |
| **LC NA cell density (count/mm^2^)** | **-0.479** | **0.022** |  | -0.142 | 0.286 |
| **LC NA fiber load (%)** | -0.259 | 0.150 |  | 0.043 | 0.433 |
| **M1 DBH+ load (%)** | -0.169 | 0.251 |  | 0.162 | 0.260 |
|  |  |  |  |  |  |
| **PD** | **M1 tract FA** | |  | **M1 tract MD (10^-3^ mm^2^/s)** | |
|  | Pearson's r | p-value |  | Pearson's r | p-value |
| **LC NA cell density (count/mm^2^)** | **-0.518** | **0.063** |  | -0.021 | 0.477 |
| **LC NA fiber load (%)** | -0.210 | 0.280 |  | 0.100 | 0.391 |
| **M1 DBH+ load (%)** | -0.197 | 0.293 |  | 0.304 | 0.196 |

Abbreviations: AD, Alzheimer’s Disease; PD, Parkinson’s disease; DLPFC, dorsolateral prefrontal cortex; M1, primary motor cortex. FA, fractional anisotropy; MD, mean diffusivity; DBH, dopamine-beta hydroxylase; mm, millimeter; r, rho.

**Supplementary materials for the script of LC meta mask registration using Advanced Normalization Tools (ANTs, version 2.1):**

/path/to/antsRegistration -n 8 --dimensionality 3 --float 0 --output SubjectT1_MNIavg152_ --interpolation Linear \

--winsorize-image-intensities [0.005,0.995] \

--use-histogram-matching 1 --initial-moving-transform [ /path/to/avg152T1_brain.nii.gz , SubjectT1.nii.gz , 1] \

--transform Rigid[0.1] \

--metric MI[ /path/to/avg152T1_brain.nii.gz , SubjectT1.nii.gz , 1,32,Regular,0.25] \

--convergence [1000x500x250x100,1e-6,10] \

--shrink-factors 8x4x2x1 \

--smoothing-sigmas 3x2x1x0vox \

--transform Affine[0.1] \

--metric MI[ /path/to/avg152T1_brain.nii.gz , SubjectT1.nii.gz ,1,32,Regular,0.25] \

--convergence [1000x500x250x100,1e-6,10] \

--shrink-factors 8x4x2x1 \

--smoothing-sigmas 3x2x1x0vox \

--transform SyN[0.1,3,0] \

--metric CC[/path/to/avg152T1_brain.nii.gz, SubjectT1.nii.gz ,1,4] --convergence [100x70x50x20,1e-6,10] \

--shrink-factors 8x4x2x1 \

--smoothing-sigmas 3x2x1x0vox --v 1

/path/to/antsApplyTransforms -d 3 -r /path/to/avg152T1_brain.nii.gz -i SubjectT1.nii.gz -e 0 -n Linear -t SubjectT1_MNIavg152_1Warp.nii.gz -t SubjectT1_MNIavg152_0GenericAffine.mat -o SubjectT1_MNIavg152.nii.gz -v 1 > ApplySyNCoreg_01.log 2>&1

/path/to/antsApplyTransforms -d 3 -r SubjectT1.nii.gz -i /path/to/LCmetaMask_MNI05_s01f_plus50.nii.gz -e 0 -n NearestNeighbor -t SubjectT1_MNIavg152_1InverseWarp.nii.gz -t [SubjectT1_MNIavg152_0GenericAffine.mat,1] -o LCmetaMask_SubjectT1.nii.gz -v 1 > ApplySyNCoreg_02.log 2>&1

References

1. Bogerts B. A brainstem atlas of catecholaminergic neurons in man, using melanin as a natural marker. J Comp Neurol. 1981;197(1):63-80.

2. Theofilas P, Ehrenberg AJ, Dunlop S, Di Lorenzo Alho AT, Nguy A, Leite REP, et al. Locus coeruleus volume and cell population changes during Alzheimer's disease progression: A stereological study in human postmortem brains with potential implication for early-stage biomarker discovery. Alzheimers Dement. 2017;13(3):236-46.

3. Ohm DT, Peterson C, Lobrovich R, Cousins KAQ, Gibbons GS, McMillan CT, et al. Degeneration of the locus coeruleus is a common feature of tauopathies and distinct from TDP-43 proteinopathies in the frontotemporal lobar degeneration spectrum. Acta Neuropathol. 2020;140(5):675-93.

4. Braak H, Del Tredici K, Rub U, de Vos RA, Jansen Steur EN, Braak E. Staging of brain pathology related to sporadic Parkinson's disease. Neurobiol Aging. 2003;24(2):197-211.
